# Phototunable chip-scale topological photonics: 160 Gbps waveguide and demultiplexer for THz 6G communication

**DOI:** 10.1038/s41467-022-32909-6

**Published:** 2022-09-15

**Authors:** Abhishek Kumar, Manoj Gupta, Prakash Pitchappa, Nan Wang, Pascal Szriftgiser, Guillaume Ducournau, Ranjan Singh

**Affiliations:** 1 Division of Physics and Applied Physics, School of Physical and Mathematical Sciences, Nanyang Technological University, Singapore, Singapore; 2 Centre for Disruptive Photonic Technologies, The Photonics Institute, Nanyang Technological University, Singapore, Singapore; 3 Institute of Microelectronics, Agency for Science, Technology and Research (A*STAR), Singapore, Singapore; 4 Laboratoire de Physique des Lasers, Atomes et Molécules (PhLAM UMR 8523), Villeneuve d’Ascq CEDEX, France; 5 Université de Lille, CNRS, Centrale Lille, Univ. Polytechnique Hauts-deFrance, UMR 8520, IEMN—Institut d’Electronique de Microélectronique et de Nanotechnologie, Lille, France

**Keywords:** Integrated optics, Terahertz optics

## Abstract

The revolutionary 5G cellular systems represent a breakthrough in the communication network design to provide a single platform for enabling enhanced broadband communications, virtual reality, autonomous driving, and the internet of everything. However, the ongoing massive deployment of 5G networks has unveiled inherent limitations that have stimulated the demand for innovative technologies with a vision toward 6G communications. Terahertz (0.1-10 THz) technology has been identified as a critical enabler for 6G communications with the prospect of massive capacity and connectivity. Nonetheless, existing terahertz on-chip communication devices suffer from crosstalk, scattering losses, limited data speed, and insufficient tunability. Here, we demonstrate a new class of phototunable, on-chip topological terahertz devices consisting of a broadband single-channel 160 Gbit/s communication link and a silicon Valley Photonic Crystal based demultiplexer. The optically controllable demultiplexing of two different carriers modulated signals without crosstalk is enabled by the topological protection and a critically coupled high-quality (*Q*) cavity. As a proof of concept, we demultiplexed high spectral efficiency 40 Gbit/s signals and demonstrated real-time streaming of uncompressed high-definition (HD) video (1.5 Gbit/s) using the topological photonic chip. Phototunable silicon topological photonics will augment complementary metal oxide semiconductor (CMOS) compatible terahertz technologies, vital for accelerating the development of futuristic 6G and 7G communication era driving the real-time terabits per second wireless connectivity for network sensing, holographic communication, and cognitive internet of everything.

## Abstract (web summary)

6G communication requires high-speed and advanced functionalities on-chip. Here the authors demonstrate broadband phototunable topological waveguide and demultiplexing chip with record single-channel 160 Gbit/s communication link and excellent channel isolation for 300 GHz band.

## Introduction

The symbiosis of physical, digital, and biological worlds is one of the core visions of sixth-generation (6G) communication^1^, driven by various foreseen disruptive applications like industrial automation, innovative society, intelligent healthcare system, massive internet-of-everything, and energy-efficient networks. The realization of these applications critically requires ultra-high-speed connectivity with sub-millisecond latency times^2,3^. One of the tenets of the development of 6G technology is to expand the frontier of the radiofrequency (RF) spectrum into the terahertz (THz) band (beyond 300 GHz) for accessing the larger bandwidth^2,4^. As 6G wireless networks evolve, emerging communication devices are expected to handle a large volume of data, such as the real-time transmission of high resolution 8K video, paving the path towards remote healthcare and human augmentation. Thus, the system-on-chip architecture must support significant bandwidth signals to efficiently process and compute the extensive volume data to integrate seamlessly with 6G networks. High-speed on-chip communication technologies offering data rates beyond 100 Gbit/s have become crucial for emerging devices. Inter and Intra chip communication facilitated by metallic *electrical interconnects*^5,6^ becomes exceptionally lossy at THz frequencies. Alternatively, *optical interconnects*^5,6^ faces system integration complexity with additional electronic-to-optical and optical-to-electronic conversion losses. Therefore, a novel design paradigm of the *terahertz interconnect* with advanced functionalities such as on-demand active control, and channel demultiplexing is required to achieve high-speed on-chip communication. Conventional approaches of THz interconnect waveguides such as metallic transmission line^7^, dielectric strip waveguide^8^, photonic crystal waveguide^9^, and THz fibers^10^ are prone to defects, reflection/scattering loss, material dispersion, bending loss, and lack active tunability. These challenges have limited the on-chip single and multiple channel data transmission speeds to few tens of gigabits per second, which presents a significant challenge for developing 6G THz communication devices.

Here, we demonstrate a new class of broadband phototunable silicon (Si) THz topological devices consisting of single-waveguiding channel that supports data rates of up to 160 Gbit/s. In an advanced design, the on-chip topological waveguide is critically coupled to a high-quality factor topological cavity, which converts the coupled system into a topologically protected photoactive demultiplexer device that exhibits perfect isolation between resonant and non-resonant carrier frequencies. We demultiplexed spectrally efficient 40 Gbit/s quadrature amplitude modulation (QAM) signals and showed real-time streaming of uncompressed high-definition (HD) video signal (1.5 Gbit/s) using the topological waveguide-cavity chip. Our study reveals that the topological protection of edge states preserves the high-*Q* mode of the topological cavity, ensuring excellent channel isolation between the demultiplexed channels. The edge states provide robust transport of THz data signals through sharp bends and defects without scattering, which is essential for the highly efficient THz interconnects and device miniaturization. The topological chip is built based on Valley Photonic Crystal (VPC)^11–13^ that hosts valley-dependent helical edge states traveling in opposite directions within highly compact structures without the need for any magnetic materials and other complex systems. In addition, these valley edge states are single-mode and exhibit linear dispersion characteristics. The edge states also reduce the mode competition and frequency-dependent signal delay^14^ and effectively allow the entire bandwidth of the VPC waveguide to be used for on-chip communication. This has stimulated a growing interest for developing THz topological integrated circuits. Along this line, recently, on-chip THz communication was demonstrated using VPC waveguide^14,15^. However, these proof-of-concept devices exhibit limited bandwidth and lack active tunability, which is essential for integrated on-chip THz manipulation with advanced functionality. We circumvent these limitations by enhancing the usable bandwidth (~30 GHz transmission band) in our topological devices and demonstrate record value of on-chip single-channel communication link with 160 Gbit/s (32 GBaud symbol rate, BER—1.3 × 10^−2^) and 125 Gbit/s (25 GBaud symbol rate, BER—1.0 × 10^−3^) data rates using QAM-32 with straight and bent topological chips, respectively (see Tables S2, S3 in Supplementary Information). We achieve active tunability by photoexciting the Si-VPC chip, where the entire communication channel is switched ON and OFF while preserving its topological features. The phototunable VPC chip enables essential functionalities such as on-demand channel filtering^16^, switching^17^, and active beam steering^18^ and paves the path for developing high-density on-chip communication channels without crosstalk for emerging 6G communication devices.

Besides that, channel demultiplexing is a crucial technology for communication. It allows large transmission bandwidth to be simultaneously used for multiple independent signals with high data throughput in the communication network. Therefore, we designed an on-chip topological demultiplexer consisting of a VPC waveguide critically coupled to a high-*Q* topological cavity. It enables perfect isolation between two different carrier modulated signals without any crosstalk. We demonstrate demultiplexing of dual THz channels containing two differently modulated data signals: 331.6 GHz (CH 1) and 344 GHz (CH 2) as carrier frequencies. We show real-time HD-video streaming with 1.5 Gbit/s and data transmission with 40 Gbit/s from channels 1 (CH 1) and 2 (CH 2), respectively. We further verify the channel isolation by photoexcitation, where the negligible effect in HD-video streaming at CH 1 was observed even after attenuating the signal in CH 2 upon photoexcitation. Notably, the previous THz topological photonic reports^19,20^, solely discusses about the high quality (*Q*) mode of topological cavity and its optical modulation. In this work, we establish a detailed theoretical framework for dynamic critical coupling in the topological waveguide-cavity chip and demonstrate broadband THz topological waveguiding and switching (~30 GHz transmission band) with record single-channel data rate of 160 Gbit/s and an active chip-scale topological demultiplexing for 300 GHz band envisioned for future generation of 6G, and 7G wireless communication. Such an active control of topological devices will inspire a plethora of applications like on-chip modulator^21^, reconfigurable beam steering^22^, and dynamic signal routing^18^. In addition, we envision that the high-*Q* topological cavity-waveguide chip provides a robust testbed for exploring numerous phenomena such as nonlinear topological photonics^23^, topological quantum circuits^24^, ultra-low threshold topological lasers^25–28^ and topological light-matter interactions^29,30^.

## Results

**Fig. 1:**
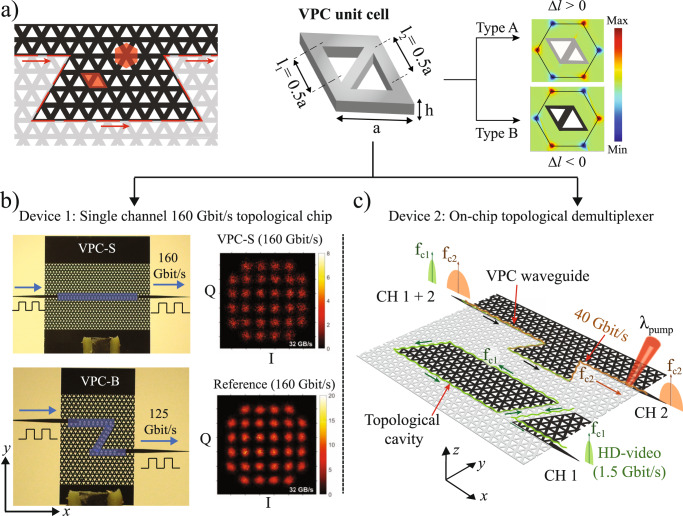
Phototunable on-chip THz topological devices for 6G communication. **a** Schematic of the topological interface showing two topologically distinct regions (black and gray regions). The domain wall is highlighted with a red dash line that hosts robust propagation of THz waves even at sharp bends. The red shaded regions show Wigner-Seitz and unit cell (rhombus-shaped). The central portion depicts the unit cell of VPC where the side of equilateral triangular air holes are marked with l_1_ and l_2_, respectively. The sign of Δ*l* = *l*_1_ − *l*_2_ differentiate the Type A (Δ*l* > 0) and Type B (Δ*l* < 0) unit cells. The right panel shows the numerically calculated distribution of Berry curvature for both the unit cells. The Berry curvature is localized near the K and K′ valley, and Type A and Type B unit cells exhibit complementary distribution. **b** Optical image of fabricated straight (VPC-S) and bend (VPC-B) Si-VPC chips. An adiabatic tapered couplers were designed at both the input and output facets to efficiently in and out couple the THz waves from Si-VPC chips. The domain wall in Si-VPC chip is highlighted with a blue shaded region. The highest data rate recorded through VPC-S and VPC-B chips is 160 Gbit/s and 125 Gbit/s, respectively. Inset shows the I–Q constellation diagram corresponding to 160 Gbit/s using QAM-32 with 32 GBaud symbol rate through the VPC-S chip and reference (without the chip). The I–Q constellation diagram through the VPC-B chip is shown in Supplementary Information Fig. S8. **c** A schematic of an on-chip topological demultiplexer. Two differently modulated data signals at carrier frequencies f_c1_ and f_c2_ were injected into VPC waveguide, further demultiplexed into two separate channels: CH 1 and CH 2. The data signal with carrier frequency f_c1_ was modulated with an ON-OFF keying scheme for sending real-time HD video at 1.5 Gbit/s to CH 1 (coupled to waveguide via cavity, green arrow), while carrier frequency f_c2_ was modulated with QAM-16 (10 GBaud symbol rate) to achieve direct data transfer rate of 40 Gbit/s at CH 2 (orange arrow). The high *Q* nature of the topological cavity ensures excellent isolation between CH 1 and 2. It is verified by photoexciting (shown as λ_pump_) the CH 2, which attenuated the 40 Gbit/s without affecting the real-time streaming of HD-video at CH 1. Please refer to the Supplementary Videos: Supplementary Movie “a” and Supplementary Movie “b” for a clear illustration of phototunable demultiplexing.

**Fig. 2:**
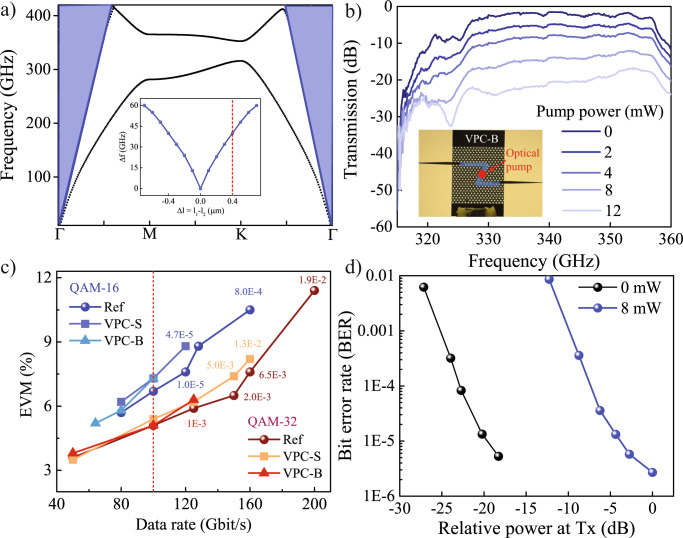
Beyond 100 Gbit/s phototunable silicon Valley-Hall photonic crystal THz waveguide. **a** Photonic band diagram calculated using COMSOL Multiphysics for Si-VPC chip with *l*_*1*_ and *l*_*2*_ equal to 0.70*a* and 0.30*a* respectively. Inset shows the variation in bandgap as a function of the difference in triangular hole size (*Δl* = *l*_*1*_ *−* *l*_*2*_). **b** Measured transmission from VPC-B chip at various photoexcitation pump power. Inset shows the optical image of the fabricated VPC-B chip where the blue region highlights the domain walls. The red dot represents the photoexcitation position at the domain wall. **c** Error vector magnitude (EVM) versus data rate for both the straight (VPC-S) and bend (VPC-B) VPC chips recorded at different modulation formats (QAM-16 and 32). All the measurements were compared with reference measurements (without the chip). The value of bit-error-rate (BER) corresponding to higher data rates are written with the corresponding data points. **d** BER versus relative power at the transmitter (Tx) recorded at 50 Gbit/s for the VPC-B chip. The black curve corresponds to the no pump (0 mW) case, while the blue curve was recorded with 8 mW photoexcitation.

The THz topological chip is made of high-resistive silicon (HR-Si) with resistivity >10 kΩ-cm. The HR-Si offers low absorption loss, large nondispersive refractive index (~3.42)^31^ and CMOS compatibility. Figure 1a shows the schematic of Si VPC, composed of a honeycomb lattice of triangular holes with a lattice constant *a* = 242.5 μm. The unit cell consists of two inverted equilateral triangular air holes of side length *l*_*1*_ and *l*_*2*_ (rhombus-shaped red shaded region in Fig. 1a). When the size of triangular holes is equal (*l*_1_ = *l*_2_ = 0.5*a*), the photonic crystal exhibits C_6_ symmetry and features a pair of degenerated Dirac points at K and K’ valleys (see Supplementary Information, Fig. S1). The inversion symmetry can be broken by setting *l*_1_ ≠ *l*_2_, which reduces the photonic crystal structure to a C_3_ symmetry that lifts the degeneracy of Dirac points and opens the topological bandgaps at K and K′ valleys (see Figs. 2a and S1). Here, we focus on the transverse electric (TE) modes for which electric fields are confined within the plane (*xy* plane). We define Δ*l* = *l*_1_ − *l*_2_ to differentiate the Type A (Δ*l* > 0) and Type B (Δ*l* < 0) unit cells shown by gray and black color in Fig. 1a, respectively. Since the photonic crystal possesses the time-reversal symmetry, the integration of Berry curvature (an abstract ‘magnetic field’ in the momentum space) over the entire Brillouin zone vanishes. However, for a given unit cell the Berry curvature is localized near K and K′ valleys (see Fig. 1a) with opposite signs. Therefore, integrating the Berry curvature near K/K’ valley results in a nonzero Chern number, called valley-Chern number which is a *topological invariant*^11^. Since the Type A and Type B unit cells exhibit complementary Berry curvature distribution, integration of the Berry curvature around K/K′ valley yields opposite signed valley-Chern numbers that denotes different topological phases^11–13,32^. Interfacing the Type A and Type B unit cells form domain walls where a pair of valley-polarized topological edge states exist due to bulk-boundary correspondence^33^. The propagation direction of these edge states is locked to their valleys and exhibit robust transport of electromagnetic waves even at sharp corners or bends. To highlight the robustness, we designed our THz topological devices with sharp bends, as highlighted in Fig. 1b, c. Here, we considered two types of devices: Device 1, which includes the straight (VPC-S) and bend (VPC-B) VPC waveguide chips, and Device 2 is an on-chip topological demultiplexer. Figure 1b shows the optical images of fabricated VPC-S and VPC-B chips. The domain walls of VPC chips are highlighted with blue shaded rectangles in Fig. 1b. We demonstrated a single-channel communication link with a data rate of 160 Gbit/s and 125 Gbit/s through VPC-S and VPC-B chips, respectively. The reduced data rate for the case of the VPC-B chip is discussed in the later part of the paper. The inset of Fig. 1b shows the I–Q constellation diagram recorded for 160 Gbit/s data rate using QAM-32 with 32 GBaud symbol rate for both the VPC-S chip and reference (without the chip). The well-isolated constellation points represent negligible distortion in amplitude and phase of transmitted signal, which signifies the robust transport of data signals through VPC chip.

Figure 1c shows the schematic of an on-chip topological demultiplexer. It consists of a VPC waveguide critically coupled to a topological cavity with a high *Q* factor. The distance between waveguide and cavity is optimized to achieve maximum coupling efficiency, enabling frequency demultiplexing of single channel (CH 1 + 2) into two well-isolated channels (CH 1 and CH 2) with negligible crosstalk. Figure 1c depicts the frequency demultiplexing functionality where the input channel (CH 1 + 2) carrying dual THz channels with data rate 1.5 Gbit/s and 40 Gbit/s at carrier frequencies f_c1_ (331.6 GHz) and f_c2_ (344 GHz) respectively is demultiplexed into CH 1 (shown by the green arrow) and CH 2 (shown by the orange arrow). Photoexciting CH 2 (shown as λ_pump_ in Fig. 1c) attenuates the 40 Gbit/s data signal (see Fig. 1c) without affecting the real-time HD-video streaming at CH 1. To realize the on-chip topological demultiplexer, the topological cavity is designed by forming a close looped domain wall between Δ*l* > 0 (gray color) and Δ*l* < 0 (black color) unit cells (see Fig. 1c). Despite the sharp corners in the topological cavity, the edge state circulates with negligible back reflection and give rise to regularly spaced discrete eigenmodes. The topological protection of these edge states preserves the high-*Q* modes of the topological cavity that ensure excellent isolation between the demultiplexed channels. In this sense, the topological cavity is unique compared to conventional photonic cavities such as nanobeam cavity^34^ and photonic crystal cavity^35,36^ whose resonance modes are extremely sensitive to small structural imperfection and induces significant scattering loss.

### Phototunable broadband topological switch

The Si-VPC chip was designed to achieve large bandwidth. Tuning the size of triangular holes in the unit cell alters the topological bandgap by changing the effective refractive index contrast between the Si slab and surrounding medium. The inset of Fig. 2a shows the bandgap variation as a function of the difference in triangular holes size defined as Δ*l* = *l*_1_ − *l*_2_. Due to limitations imposed by fabrication constraints, we designed the Si-VPC chip with triangular holes of side length *l*_1_ = 0.7*a* and *l*_2_ = 0.3*a* corresponding to Δ*l* = 0.4*a* (vertical red dotted line in inset Fig. 2a) that enable ~30 GHz of transmission band. This is further verified by the photonic band diagram calculated using COMSOL Multiphysics (see the Methods and Supplementary Information, Section S1) shown in Fig. 2a. To experimentally demonstrate the broadband feature, we performed THz transmission measurement for the fabricated Si-VPC chip, and the results are shown in Fig. 2b. The inset of Fig. 2b shows the optical image of the fabricated VPC-B chip with domain wall highlighted by the blue shaded region. The transmission measurements were performed using a vector-network analyzer (VNA) based setup. The readers are directed to the Method and Supplementary Information Section S6 for the detailed discussion of the measurement setup. The measured transmission spectrum shows the distinct bandgap extending from 325 to 355 GHz (see Fig. 2b). The experimentally recorded bandwidth (Δ*f* ~30 GHz, transmission band) is lower than the simulation, which may be due to small inhomogeneity in the size of triangular holes in the fabricated chip. Furthermore, to achieve active control, the Si-VPC chip was photoexcited at the domain wall (see inset of Fig. 2b) with a 780 nm (E = 1.59 eV) optical pump beam from an external cavity semiconductor laser. Photoexciting the Si above the bandgap (E > E_g_ ~1.1 eV^37^) generates free carriers that modulate the THz waves by adding a strong attenuation within the waveguiding path. The modulation depth for the entire bandgap region increases with increasing pump power and subsequently features >25 dB attenuation for 12 mW pump power with a circular spot size of ~1 mm diameter. Here, it should be noted that the photoexcitation does not destroy the topological protection in the VPC-B chip, thus, maintain the flat transmission curve for each pump power (see Fig. 2b). This acts as a broadband THz topological switch where the entire communication channel can be switched OFF and ON, with a flat transmission curve enabling wideband modulated channels in the 300 GHz band. Figure S5.2 shows the ON/OFF switching measurement where we achieved up to 1 MHz switching speed mainly limited by the slow carrier relaxation time in Si. The switching speed could be further enhanced to gigahertz through ion implantation^31,38^ of Si-VPC chip.

The valley edge states in the VPC chip can be excellent information carriers for on-chip communication owing to its robustness against sharp bends, linear dispersion, and single-mode propagation. To highlight the potential application of broadband Si-VPC chip, we performed a THz communication experiment at carrier frequency 340 GHz for both the VPC-S and VPC-B chips. To quantify the communication results, the error vector magnitude (EVM) as a function of data rate is plotted for both the VPC-S and VPC-B chips, as shown in Fig. 2c. EVM is a performance indicator of the demodulated I–Q map, quantifying the quality of the received signal. The presence of system imperfections in terms of phase noise, broadband thermal noise, linear distortion (system response and filtering) and non-linearity can contribute to an increase of the EVM with increasing data rates. Therefore, even without the VPC chip (reference measurement), we observed an increase in EVM at a higher data rate, as shown in Fig. 2c (spherical dots). The increment in EVM value with increasing data rates occur due to the THz communication system’s limitations (not a limitation of the Si-VPC chip). It provides a reference for us to quantify the performance of VPC chip. We observed continuous increase in EVM with increasing data rate for all the three cases: reference, VPC-S and VPC-B chips as shown by spherical, square, and triangular dots, respectively in Fig. 2c.

Interestingly, for lower data rate (<100 Gbit/s), highlighted by the red vertical dotted line, the performance of the bend VPC-B chip exceeds that of the straight VPC-S chip. The is due to the presence of bends in the VPC-B chip, which mitigates the reflected signals, thus reducing the noise in the system. Ideally, the VPC-B chip must exhibit better performance for all the data rates. However, in our measurement, we observe the performance of the VPC-S chip to be slightly better than the VPC-B chip for extremely large data rates. It arises due to the appearance of an additional dip in the transmission spectrum for the VPC-B chip at 325 GHz, as shown in Figs. S9 and  2b. Also, the presence of additional dip effectively reduces the bandwidth of the VPC-B chip compared to the VPC-S (see Fig. S9) chip. It limits the highest data rate for the VPC-B chip to be 125 Gbit/s using QAM-32 (25 GBaud symbol rate) with bit-error-rate (BER) 1.0 × 10^−3^ (see the red triangular dot, Fig. 2c). The careful design of the coupler can eliminate the additional dip by efficiently coupling the lower frequencies onto the VPC-B chip with bends.

Moreover, with QAM-32 (32 GBaud symbol rate), we achieved a data rate of 160 Gbit/s with BER 1.3 × 10^−2^ (see the orange square dot, Fig. 2c) through the VPC-S chip. The I–Q intensity constellation diagram for recovered symbols through the VPC-S chip and reference (without the chip) is shown in the inset of Figs. 1b,  S8. The clearly defined constellation points show sufficient signal-to-noise ratio (SNR) level, system fidelity and good performance of software-based digital-signal processing (DSP) equalization. The I–Q constellation diagram for 125 Gbit/s using QAM-32 (25 GBaud) through VPC-B chip and the detailed discussion on the communication measurement setup are presented in Supplementary Information Section S7.

Furthermore, to quantify the effect of photoexcitation on communication performance, we assessed the BER performance in the VPC-B chip (see Fig. 2d). The higher-order modulation enhances the data rate in the waveguiding channel as well as the spectral efficiency. The channel path loss is an important parameter in determining the requirements of transmitted power for maintaining optimum BER performance. Figure 2d shows the variation in BER values for QAM-16 modulation format with relative THz transmitted power (at 340 GHz) in communication experiments. An increase in transmission power increases the SNR, reducing BER and improving channel performance. Here, as photoexcitation induces a loss in the waveguiding channel of the VPC-B chip, SNR is reduced. Therefore, higher transmitted power is required to improve the SNR for maintaining the same BER value. The BER decreased linearly (black curve) with an increase in transmitted power propagating in the VPC-B chip without the presence of photoexcitation. However, the photoexcitation of the domain wall with 8 mW of pump power with beam spot size ~1 mm diameter shifts the entire curve to higher values of the transmitted power to maintain the same BER characteristics (blue curve, Fig. 2d). The power penalty (power shift between the two curves) observed in Fig. 2d corresponds to the attenuation of THz waves (due to photoexcitation) in the VPC-B chip measured using the VNA. Importantly, the slope of the curve remains the same, indicating that no additional distortion or bandwidth reduction was recorded, thus topological protection of THz waves is ensured even with the generation of photoexcited carriers within the waveguiding path.

### Phototunable topological demultiplexing chip

**Fig. 3:**
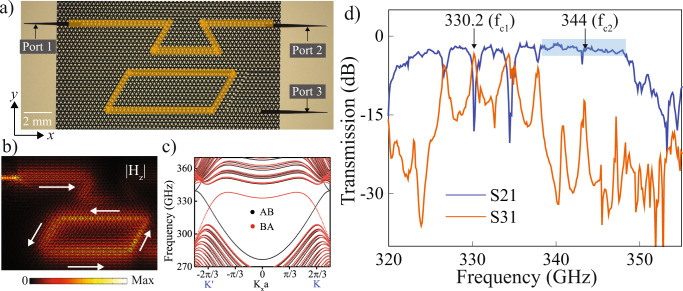
Coupled topological waveguide-cavity as an on-chip demultiplexer. **a** Optical image of the fabricated VPC cavity-waveguide chip. Orange lines indicate the domain walls corresponding to VPC waveguide and topological cavity, respectively. **b** The simulated magnetic field intensity distribution of valley edge state at the cavity resonance frequency (330.2 GHz). The white arrows indicate the Poynting vectors for the edge states. **c** Projected band diagram for the supercell representing both AB (black dot) and BA (red dot) type domain walls. **d** Experimentally recorded transmission spectra at ports 2 and 3 through Si-VPC chip. The transmission spectrum corresponding to port 2 (S21) is shown in the solid blue line, while port 3 (S31) is shown with a solid orange line. The carrier frequency corresponding to channel 1 (CH 1) and channel 2 (CH 2) are marked as f_c1_ = 330.2 GHz and f_c2_ = 344 GHz, respectively. The blue shaded region in S21 represents the bandwidth (~14 GHz) corresponding to CH 2.

Due to the broad bandwidth of the Si-VPC chip (as shown in Fig. 2a, b), multiple channels become available and require a channel demultiplexing technique to utilize the full capacity of the available bandwidth. We designed the THz channels with demultiplexing functionality by integrating a topological cavity with the VPC waveguide. Figure 3a shows the optical image of the topological cavity integrated with the VPC waveguide where both the input and output couplers are used to efficiently in and out couple the THz waves from the Si-VPC chip, respectively. The domain wall corresponds to the VPC waveguide, and the topological cavity are highlighted by orange shaded lines. Figure 3b shows the simulated intensity distribution of the magnetic field at the cavity resonance frequency (330.2 GHz). The calculated Poynting vectors (white arrows) indicate that the waves propagate across the sharp bends with negligible back-reflection. Notably, they propagate in opposite directions in the waveguide and cavity, which can be explained by the fact that these are formed by different types of domain walls (AB and BA, respectively). As shown in the projected band diagram in Figs. 3c and  S2, the AB and BA domain walls host edge states of opposite group velocity for each valley. Figure 3d depicts the transmission spectra recorded at both the output ports (port 2 and 3). The blue and orange solid lines represent the transmission spectra recorded at ports 2 and 3 and referred to as S21 and S31, respectively, later in the paper.

**Fig. 4:**
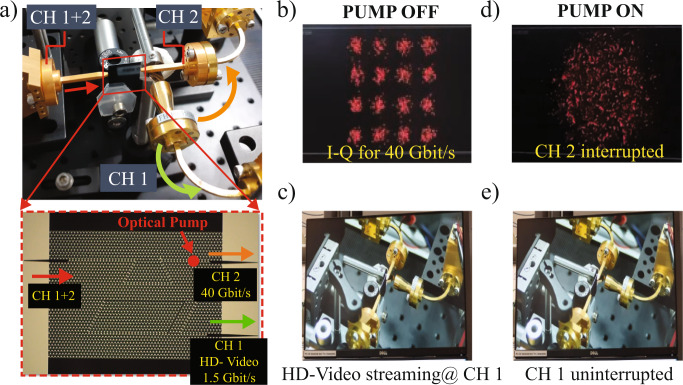
HD-video streaming and perfect channel isolation in photoactive topological demultiplexer. **a** THz communication experimental setup. Here two signals at different carrier frequencies (331.6 GHz and 344 GHz) were injected at the input port (highlighted as CH 1 + 2) of the Si-VPC chip. Due to the limited spacing between CH 1 and CH 2 couplers in the Si-VPC chip (shown in inset), we used a parabolic mirror to redirect the signal from CH 1 and collected it by using a horn antenna (**a**). Inset shows the optical image of fabricated Si-VPC chip with one input and two output ports. The red arrow highlights the input channel (CH 1 + 2), which further demultiplexed into two channels: CH 1 (green arrow) and CH 2 (orange arrow). **b**, **d** I–Q intensity constellation diagram recorded at channel 2 (CH 2; carrier frequency 344 GHz) of Si-VPC chip for PUMP OFF and ON case, respectively. Without photoexcitation (PUMP OFF case), we observe a clear constellation diagram corresponding to 40 Gbit/s data rate using QAM-16 with 10 GBaud symbol rate. With photoexcitation (PUMP ON case), the THz waves get attenuated. We observe fading in the constellation diagram. **c**, **e** Snapshot taken from real-time HD-video streaming transmitted via channel 1 (CH 1; carrier frequency 331.6 GHz) for pump OFF and ON case, respectively. The real-time streaming of HD video was uninterrupted even after attenuating the THz signal at CH 2 upon photoexcitation. This exhibits excellent isolation between the demultiplexed channels owing to the high-*Q* nature of the topological cavity. Please refer to the Supplementary Videos (Supplementary Movie “a” and Supplementary Movie “b”) to clearly illustrate THz channel demultiplexing.

The S21 (S31) exhibits sharp dips (peaks) at 330.2 and 334.4 GHz. These correspond to the eigenfrequencies of the topological cavity. The detailed discussion on the eigenvalue of the topological cavity is given in Supplementary Information, Section S4. The transmission spectrum, S21, reveals that the resonance modes exhibit different *Q* factors. The differences in *Q* factor arise due to different radiative and coupling losses of each resonant mode from the waveguide to the cavity. Moreover, we highlight that the *Q* factors for S31 resonances are slightly less than S21 as resonance modes at port 3, which undergoes multiple couplings (waveguide to the cavity and then cavity to the waveguide). Owing to the high *Q* nature of the cavity resonance at 330.2 GHz, we utilized this resonance mode for channel demultiplexing. To demonstrate the on-chip channel demultiplexing, we simultaneously sent two independently modulated data signals at carrier frequencies 331.6 GHz and 344 GHz (far from cavity resonance) from port 1 and retrieved from ports 2 and 3. Note that the signal from CH 1 was extracted using a parabolic mirror due to the limited spacing between the CH 1 and CH 2 couplers (see inset Figs. 4a and  S11). The presence of parabolic mirror leads to the formation of standing waves due to this the optimal operating frequency required to stream real-time HD video from CH 1 was slightly shifted from the expected value (330.2 GHz) to 331.6 GHz, corresponding to a 0.4% relative shift. (See detailed discussion in Supplementary Information, Section S9).

Further, the intensity dip (peak) in S21 (S31) can be actively tuned by bringing the topological waveguide-cavity chip into the critical coupling regime. Critical coupling^39–41^ is the condition when the total loss of the cavity becomes equal to the waveguide-cavity coupling loss that manifests as enhanced dip/peak in the transmission spectrum. Here, we attain the critical coupling condition by photoexciting the domain wall of the topological cavity. The attainment of critical coupling exhibits intensity enhancement of the topological cavity resonance, shown in Fig. S19. Critical coupling is vital for data communication, as it provides a new modality for channel switching, filtering and demultiplexing applications. We present a detailed theoretical discussion to dynamically tune the critical coupling in the topological waveguide-cavity chip in Supplementary Information, Section S10, S11. Note that, here we experimentally investigate the attainment of critical coupling in the topological waveguide-cavity chip upon photoexcitation, their effect on the performance of on-chip data communication applications such as channel filtering and add drop filter will be attempted in the future investigations.

Figure 4a shows the experimental setup of THz demultiplexing measurement, where the input taper port of the Si-VPC chip is inserted between the dual-channel transmitters. The two outputs of the Si-VPC chip used as a frequency demultiplexer were feeding two separate receivers. One receiver (CH 1) was used to detect the amplitude modulated signal (carrying the HD-video signal). This receiver is based on direct detection for real-time signal transmission. The second receiver (CH 2) is composed of a sub-harmonic mixer enabling phase/amplitude signal detection, required for high spectral efficiency modulations (QAM). The inset of Fig. 4a shows the zoomed in image of the Si-VPC chip where we labeled the input port as channel 1 + 2 (CH 1 + 2, red arrow) and output ports are labeled as CH 1 (green arrow) and 2 (orange arrow), respectively. The data loaded at carrier frequency 331.6 GHz was modulated using ON-OFF keying, while the data for carrier frequency 344 GHz was modulated with QAM-16 (10 GBaud). The carrier frequency (344 GHz) corresponding to CH 2 was chosen far from cavity resonance to access large bandwidth of 14 GHz, as shown in Fig. 3d (blue shaded region of S21). Due to the availability of large bandwidth at CH 2, we could transmit data with 40 Gbit/s data rate. Figure 4b shows the I–Q constellation diagram corresponding to QAM-16 with 10 GBaud symbol rate with an occupied spectrum of 14 GHz. Owing to the high-*Q* nature of the topological cavity resonance, we demultiplexed the input data signal into two channels with an excellent isolation ratio. To illustrate this, we photoexcited the CH 2 and attenuated the signal as a result, we observed the fading of the constellation diagram (see Fig. 4d) while the streaming of HD-Video was uninterrupted in CH 1 (see Fig. 4e). This confirms that the demultiplexed channels have excellent isolation otherwise unwanted transmitted power from CH 2 to CH 1 would have disturbed the video streaming. Furthermore, we made short Supplementary Videos to illustrate the phototunable demultiplexing from our Si-VPC chip (Supplementary Video SVa, SVb).

## Discussion

In conclusion, we designed a versatile, phototunable, broadband topological waveguide, and demultiplexer devices on CMOS compatible all-Si platform enabling THz channel manipulation for 6G technologies. The topological protection of THz waves combined with a low-loss Si platform enable robust, energy-efficient, and broadband single-channel high-speed on-chip communication link with negligible crosstalk. Leveraging the robustness of valley edge states, we demonstrated various key functionalities, including broadband THz waveguiding, switching and demultiplexing in a simple test vehicle for 300 GHz-band envisioned for future generation 6G and 7G wireless communication. Using a central frequency at 340 GHz (fixed by available testbeds), we experimentally demonstrate a single-channel communication link with a date rate of 160 Gbit/s using QAM-32. Further, upon photoexciting the Si-VPC chip enables a broadband THz topological switch that potentially could be useful for on-chip THz modulator and active channel filter for emerging 6G technologies. To efficiently utilize the capacity of bandwidth in the communication channel, demultiplexing is crucial. Hence by integrating a high-*Q* factor topological cavity with a VPC waveguide, we demonstrate an on-chip THz topological demultiplexer. We demultiplex high spectral efficiency 40 Gbit/s signals and demonstrate real-time streaming of uncompressed HD video (1.5 Gbit/s). The high-*Q* mode of the topological cavity ensures excellent isolation between the demultiplexed channel. In addition, we present an active scheme to dynamically tune the critical coupling between the topological waveguide and cavity chip that allows for the expansion of the topological photonics into new science and technological applications. Furthermore, employing deep reinforcement learning techniques^42–44^ for the design and characterization of topological devices will broaden their applicability and advance the current theoretical understanding of topological systems. We envision that our topological cavity-waveguide VPC chip with high-*Q* resonances will significantly contribute at the technological level for development of compact and low-loss THz integrated photonic devices, which is extremely important for developing THz integrated circuits for 6G communication^45,46^. At the fundamental level, high-*Q* topological cavity-waveguide VPC chip provides a testbed for exploring numerous phenomena such as topological quantum circuits^24^, nonlinear topological photonics^23^, low threshold on-chip topological lasers^25,26^, polaritons and topological light-matter interaction^29^ that are the basis of other core functionalities required for full THz signal generation, processing, and manipulation at the chip level.

## Methods

### Sample fabrication

The VPC cavity-waveguide chip was fabricated on an 8-inch diameter high-resistivity silicon (Si) wafer (ρ > 10 kΩ.cm). The Si wafer was thoroughly cleaned and 4 μm thick silicon-di-oxide (SiO_2_) layer was deposited. Photolithography was used to define the triangular etch holes. SiO_2_ was selectively etched away, exposing the Si surface. Deep reactive ion etching of Si was carried out to etch >200 μm of Si. The photoresist and SiO_2_ layers were then completely removed. Back grinding of Si wafer was carried out for thinning down the wafer to 200 μm.

### Numerical simulation

The band structure calculations were performed using a numerical frequency domain solver (COMSOL Multiphysics). A rhombus-shaped unit cell composed of two equilateral triangular holes was designed with periodicity 242.5 μm and thickness 200 μm.﻿ Floquet boundary conditions were employed at both x and y directions. In the eigensolver analysis, we performed parametric sweep of the k -vector such that it traces the point from Γ to M, M to K and K to ﻿Γ to cover the irreducible Brillouin zone. The detailed simulation results are discussed in Supplementary Information Section S1.

### Experimental section

#### Vector-Network Analyser (VNA) based setup

The VNA setup was used for characterizing the Si-VPC chip. It is composed of a pair of frequency extenders (Z-500 type) driven by a ZVA-24 from Rodhe&Schwarz, in the WR2.2 waveguide configuration to cover the frequency band of 325-500 GHz. The WR2.2 VNA head can measure down frequency response down to 315 GHz. The system was first waveguide calibrated using a TRM (Thru, Reflect, Match) waveguide calibration procedure as per WR2.2 waveguide standards. After calibration, a set of waveguide probes were used to feed the input signal to the Si-VPC chip. The transmission curves in Fig. 3d are the relative transmission measurement between the Si-VPC chip (see Fig. S6.1b) and the probe-to-probe direct connection used as a reference (Fig. S6.1c).

#### THz communication measurement setup

The testbed used for I/Q modulations is composed of tunable laser lines at 1.55 µm, where the frequency difference fixes the operating carrier frequency. One of these laser lines is fixed (193.4 THz), and the other (193.740 THz for 340 GHz operation) is modulated using an I/Q Mach Zehnder modulator. The data is generated by an arbitrary waveform generator (AWG), enabling several Baud-rate and modulation schemes to be generated. The modulated optical signal is then coupled with the CW laser, amplified using an erbium doped fiber amplifier (EDFA) to feed the THz photo mixer (uni-traveling carrier photodiode, UTC-PD). The UTC-PD is then coupled into the Si-VPC chip using the same waveguide probe used during VNA measurement. At the output of the Si-VPC chip, the same waveguide probe is used to feed the THz receiver. This receiver comprises a sub-harmonic mixer (SHM, GaAs Schottky diodes technology) featuring 8–10 dB conversion losses in the operation band. An electronic multiplication chain pumps this SHM. The IF signal at SHM output is further amplified by a wideband 30 dB gain stage (SHF810) before detection using a fast real-time oscilloscope (70 GHz bandwidth). I/Q maps, EVM and BER evaluations are computed using the VSA software and MATLAB routines. The detailed block diagram of the THz communication setup is presented in the Supplementary Information Section S6.

### Reporting summary

Further information on research design is available in the Nature Research Reporting Summary linked to this article.

## Supplementary information

Supplementary Information

Description to Additional Supplementary Information

Supplementary Video a

Supplementary Video b

Supplementary Video c

Reporting Summary

## Data Availability

The authors declare that all the data supporting the findings of this study are openly available in NTU research data repository DR-NTU at 10.21979/N9/5FK01V. Additional information related to this paper is available from the corresponding author, R.S., upon reasonable request.
