# Supplementary material for: Phototunable chip-scale topological photonics: 160 Gbps waveguide and demultiplexer for THz 6G communication
Source: Nat Commun. 2022 Sep 15;13:5404. doi: 10.1038/s41467-022-32909-6 (PMC9478161; doi:10.1038/s41467-022-32909-6)
Supplement: Supplementary file 1 — Supplementary Information [file 41467_2022_32909_MOESM1_ESM.pdf]

Phototunable chip-scale topological photonics: 160 Gbps waveguide and demultiplexer for THz 6G communication

Abhishek Kumar[1,2†], Manoj Gupta[1,2 †], Prakash Pitchappa[3], Nan Wang[3], Pascal Szriftgiser[4], Guillaume Ducournau[5], Ranjan Singh[1,2] *

[1]*Division of Physics and Applied Physics, School of Physical and Mathematical Sciences, Nanyang Technological University, Singapore 637371*
[2]*Center for Disruptive Photonic Technologies, The Photonics Institute, Nanyang Technological University, Singapore 639798*
[3]*Institute of Microelectronics, Agency for Science, Technology and Research, 2 Fusionopolis Way, Singapore 138634*

[4]*Laboratoire de Physique des Lasers, Atomes et Molécules, (PhLAM), UMLR CNRS 8523, Villeneuve d'Ascq CEDEX, France*

[5]*Institut d'Electronique de Microelectronique et de Nanotechnologie (IEMN), UMR CNRS 8520, Universite de Lille 1,59652 Villeneuve d'Ascq CEDEX, France*

* *Corresponding Author-* ranjans@ntu.edu.sg

† These authors contributed equally

Supplementary Information

S1: Band structure of Valley Photonic Crystal (VPC):

The photonic band structure of Valley Photonic Crystal (VPC) was calculated using COMSOL Multiphysics. We used an *eigenvalue solver* to calculate the eigenfrequencies for the unit cell. The unit cell of VPC consists of two inverted equilateral triangular holes with side lengths l_1 and l_2 as shown in Fig. 1a (main text of manuscript). We applied Floquet boundary conditions in both the *x* and *y* directions. In the eigenvalue solver analysis, we performed the parametric sweep to vary the *k*- vector from Γ to M, M to K and K to Γ to cover the irreducible Brillouin zone (IBZ), as shown by orange shaded region in the inset of Fig S1a. In COMSOL, to numerically calculate the eigenfrequencies corresponding to each *k* points, we defined a single parameter, k, which varies from 0 to 3. The value of 'k' from 0 to 1 defines the wave number spanning the Γ to M, 1 to 2 defines a wave number spanning the M to K edge, and 2 to 3 defines a wave number spanning the diagonal K to Γ edge of the IBZ (orange shaded region of the inset of Fig. S1a).

For the symmetric structures ($l_1 = l_2 = 0.5a$; $a = 242.5$ μm), the photonic band diagram reveals a Dirac point at K/K' valley as shown in Fig. S1a. Breaking the inversion symmetry by setting

$l_1 = 0.70a$ and $l_2 = 0.30a$, opens a topological bandgap as shown in Fig. S1b. Inset shows the schematic of Type A and Type B unit cells.

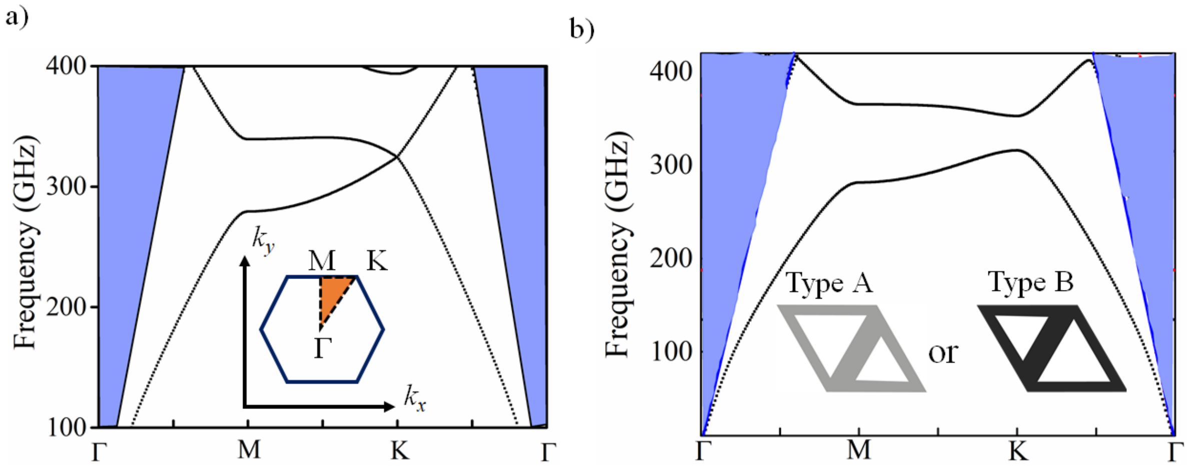

Fig. S1: Photonic band structure of VPC (a) The photonic band structure for symmetric VPC where the side length of the triangular holes is the same $l_1 = l_2 = 0.5a$; $a = 242.5$ μm, where a is the lattice constant. **(b)** Band structure after breaking the inversion symmetry by setting $l_1 = 0.70a$ and $l_2 = 0.30a$. Inset shows the schematic of Type A and B unit cells.

S2: Valley edge states propagation

Interfacing the two topologically distinct regions (here, Type A and Type unit cell) form a domain wall. There exists a pair of counterpropagating edge states at the domain wall due to bulk boundary correspondence. These edge states are locked to the valley, therefore, exhibits robust propagation against sharp bends and defects unless intervalley scattering does not take place. In our VPC waveguide-cavity chip, the propagation direction of edge states can be understood by the valley-locking analogy. The interface (domain wall) of the VPC waveguide and topological cavity are AB and BA type, respectively as shown in Fig. S2b, highlighted by the orange shaded region. Fig. S2a shows the projected band diagram of AB and BA interfaced supercell. Since the group velocity can be written as $v_g = \frac{d\omega}{dk}$,therefore, from the projected band diagram, it is evident that for a given interface (AB or BA), the propagation direction of edge states is opposite at K and K'. And for a fixed valley (K or K'), the propagation direction of edge states is opposite for AB and BA typed domain walls. Fig. 2c in the main text of the manuscript depicts the simulated intensity distribution at cavity resonance frequency which clearly shows the propagation direction in VPC waveguide and topological cavity are opposite. This also confirms that the integration of cavity with VPC waveguide does not destroy the topological protection of valley edge states.

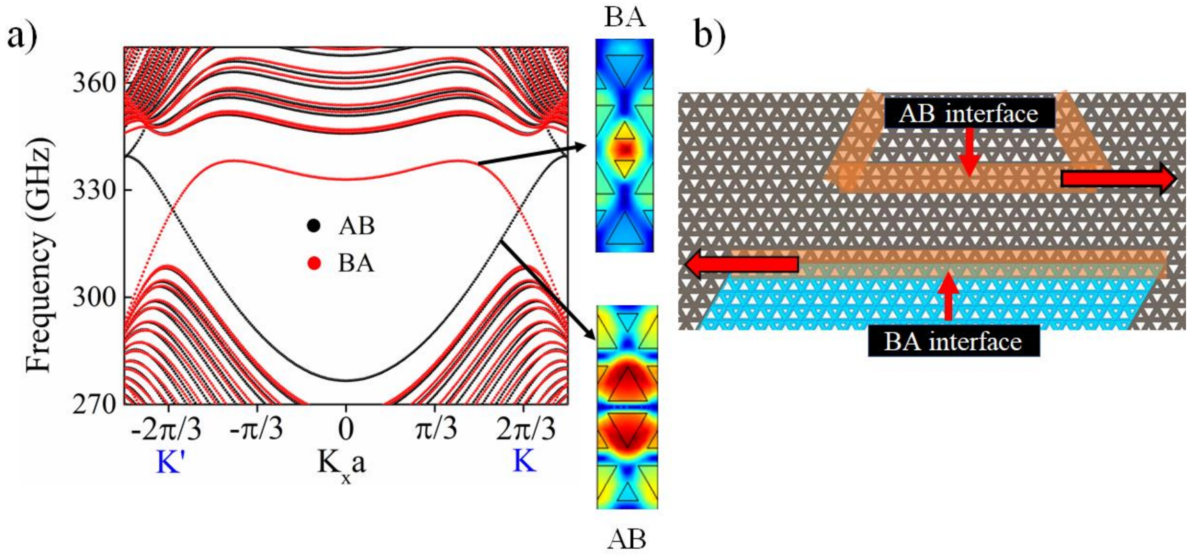

Fig. S2: Propagation direction of edge states in VPC chip. a. Simulated projected band diagram for AB and BA interfaced domain wall. The group velocity $v_g = \frac{d\omega}{dk}$ has opposite signs for AB and BA interfaced, as can be seen in the projected band diagram. Inset shows the simulated electric field distribution, which depicts that the electric fields are confined to the domain wall. **b.** Schematic of VPC waveguide-cavity integrated chip where the domain walls are highlighted with the orange shaded region. The arrows depict the propagation direction of edge states at AB and BA type domain walls.

S3: Transmission spectra from Si-VPC chip

To select the demultiplexing channel, we analysed the transmission spectra from the Si-VPC chip, as shown in Fig. S3. The sharp dips observed in S21 corresponds to the eigenfrequencies of the topological cavity. Since the edge states undergo multiple couplings (waveguide to cavity and cavity to waveguide, see Fig. 3a) for port 3 hence the resonance *Q* factor for S31 is less compared to S21. To extract the data signal from port 3 (i.e., CH 1), we used an external parabolic mirror due to the limited space between port 2 and port 3 coupler (see inset of Fig. 4a). This leads to the formation of standing waves because of this, the optimal operating frequency required to stream real-time HD video was slightly shifted from the expected value (330.2 GHz) to 331.6 GHz, corresponding to a 0.4% relative shift. Hence, we selected 331.6 GHz (f_{c1}) as a carrier frequency for CH 1 and 344 GHz (f_{c2}) for CH 2. The available bandwidth for CH 2 is larger than CH 1, which is ~ 14 GHz (a blue highlighted region in Fig. S3a). Hence, we used QAM-16 modulation format with a 10 GBaud symbol rate to transmit data with 40 Gbit/s through CH 2. Meanwhile, the available bandwidth for CH 1 was sufficient to transmit data for real-time streaming of HD-Video. A detailed discussion regarding the demultiplexing measurements are presented in section S9.

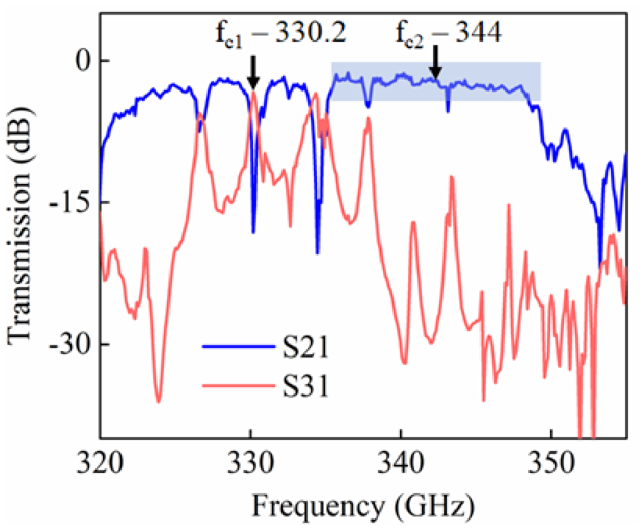

Fig S3: Transmission spectra from Si-VPC chip. Experimentally recorded transmission from both the output ports of VPC chip. S21 (solid blue line) and S31 (solid orange line) represent the transmission corresponding to ports 2 and 3, respectively. For demultiplexing, we select the carrier frequencies to be well separated from each other to reduce spectral overlap. The high Q nature of the topological cavity further helps to reduce the crosstalk between the demultiplexed channels.

S4: Eigenvalue analysis of topological cavity

The topological edge states exist at the interface of two domains with topologically distinct photonic band structures. Forming a close loop by interfacing Type A and Type B domains creates a topological cavity where the cavity modes reside at the interfaces with topological protection. Fig. S4a shows the schematic to realize the topological cavity. The robustness of the edge states allows the resonance modes to exist even at the presence of sharp corners (see Fig. S4b). Due to finiteness of the topological cavity, there exists discrete eigenmodes within the photonic bandgap[1,2]. The occurrence and number of eigenmodes is highly correlated with the topological cavity size. The topological cavity can support two types of resonant modes viz. a) ring-resonator or whispering gallery modes (WGMs) and b) standing wave modes (SGMs) as discussed in several previous reports[1,2] as well. Since we intend to realize demultiplexing functionalities using the topological waveguide-cavity system, we focus on utilizing the WGMs due to its running mode characteristics. The intrinsic nature of both the WGM and SGM modes are different, therefore, the condition to achieve critical coupling for these two different resonating modes would be different[2]. The detailed discussion on achieving critical coupling in our topological waveguide-cavity chip are discussed in section S10.

To have a resonance for traveling WGM modes in the topological cavity, an integer number of wavelengths must fit around the circumference of the topological cavity. This condition can be expressed as:

$$\lambda_m m = 2\pi n_{eff} L \qquad (S1)$$

with m representing the mode number, n_{eff} being the mode's effective index, λ_m is the resonance wavelength and L represents the length of the topological cavity.

Fig. S4b shows the numerically calculated eigenmodes of the parallelogram-shaped topological cavity. It consists of 26 x 11-unit cells. The eigenvalue is calculated using an *eigenvalue solver* in COMSOL Multiphysics. We defined a finite geometry where the parallelogram-shaped Type B unit cell is surrounded by a sufficient large Type A structure. The overall structure is surrounded by a pre matching layer (PML) layer. To emulate the 3D structure of the topological cavity from 2D simulation, we assigned the effective refractive index of silicon to 3.0. Fig. S4b shows the calculated eigenvalue. Here, we observed nearly degenerated eigenmodes. These modes correspond to clockwise and counter clockwise rotating eigenmodes. Inset shows the intensity distribution of *z*-component of magnetic field calculated at corresponding eigenfrequency of the topological cavity. The result suggests that the fields are confined through the closed-looped domain wall and exist even in sharp bends.

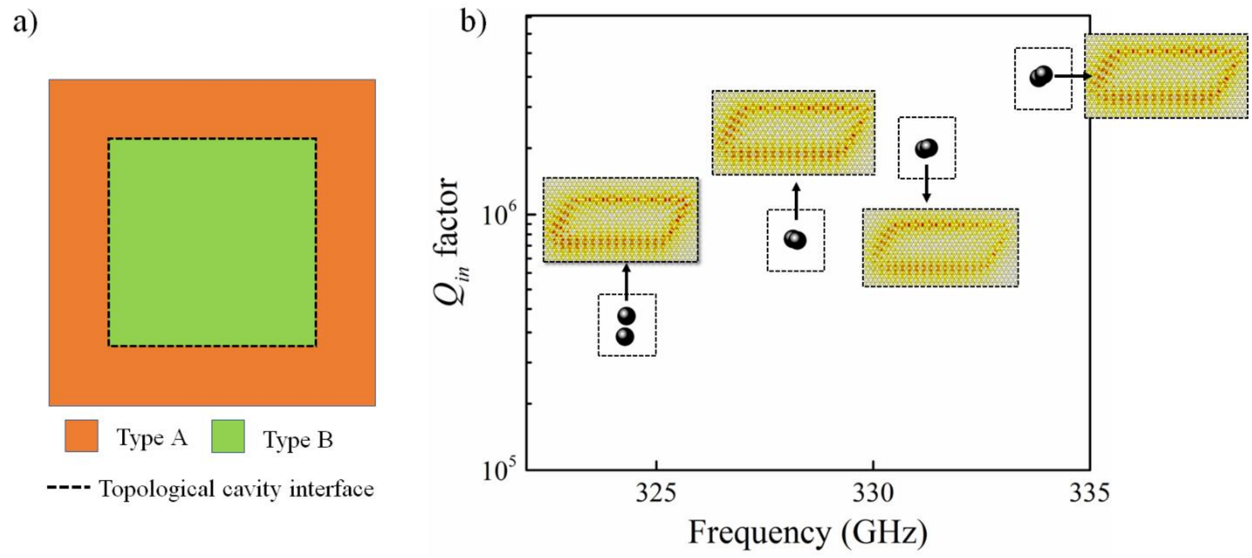

Fig. S4: a. Topological cavity is realized by forming a close loop of Type A unit cell with Type B unit cell as highlighted with orange and green colour respectively. The interface forms a domain wall where exist a pair of counterpropagating valley edge states with opposite direction of propagation. Due to this there exists degenerated modes (Fig. S4b) for each of the eigenfrequencies (see Fig. S4b). The degenerated modes correspond to the clock and counterclockwise rotating eigenmodes **b.** Calculated intrinsic *Q*-factor for eigenmodes of parallelogram shaped topological cavity. It contains 26- and 11-unit cells in the horizontal and vertical direction, respectively. Inset shows the simulated intensity distribution for *z*-component of magnetic field at corresponding eigenfrequency, which suggest that the resonant modes exist even in the presence of sharp corners of topological cavity.

S5: Switching speed of broadband Si VPC chip upon photoexcitation

The switching speed was measured using the modulation of a continuous wave (CW) signal from the VNA. We used the VNA as a source, then switched ON and OFF the laser beam (780 nm) with a controllable frequency from a few kHz to 10 MHz. Then, the THz amplitude modulated signal was detected using a spectrum analyser extension, indicated in Fig S5.1.

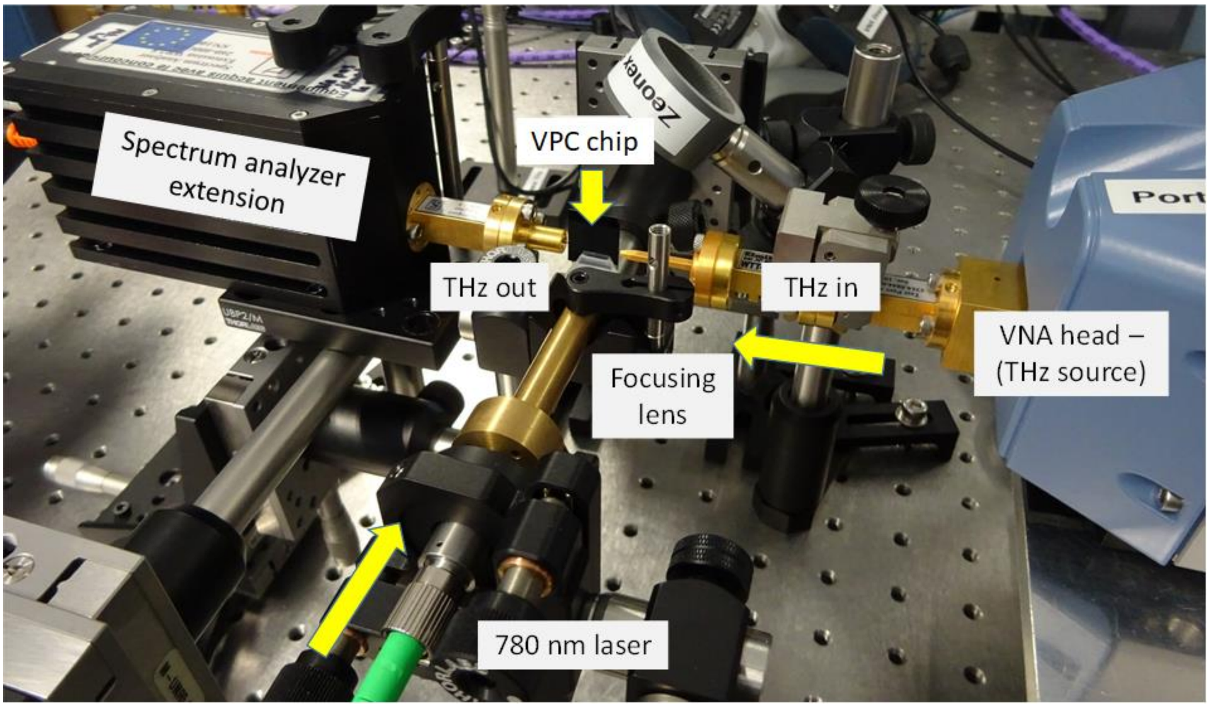

Fig S5.1. Measurement of the frequency response of the Si-VPC chip used as a switch. The VNA head (right) is used as a continuous wave (not modulated) source, 'THz in'. The Si-VPC chip is photoexcited using a 780 nm AM (amplitude modulation)-modulated (switched ON and OFF) laser beam, transferring the modulation into the THz signal propagating inside the Si-VPC chip. The output spectrum ('THz out') is then measured with the spectrum analyser extension.

Looking at the THz spectrum at Si VPC output, we observed an AM-like spectrum, on which we measured the relative evolution of the modulation lines around the THz carrier frequency. We measured the modulation lines down to the noise floor of the spectrum analyser (see Fig. S5.2).

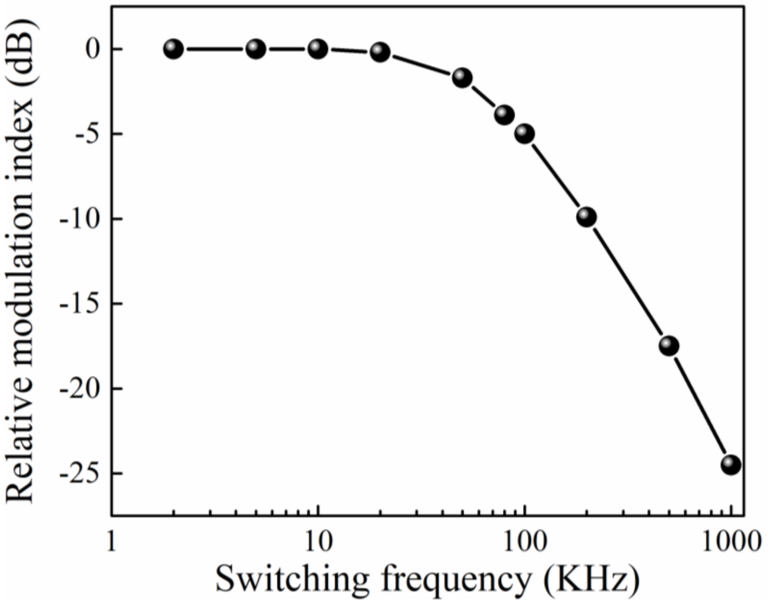

Fig S5.2: Phototunable broadband topological switch.

S6: Terahertz communication experiment setup

The VPC chips (both the VPC-S and VPC-B) and topological cavity-waveguide chip were characterized using a VNA setup comprising 2 sets of frequency extenders with operation frequency in the range of 325- 500 GHz (shown in Fig. S6.1a). WR2.2 hollow waveguide attached to VNA extenders is compatible with the coupler dimensions of the Si-VPC chip, which were used to in-couple/out-couple the signal to/from the Si-VPC chip shown in Fig. S6.1b and S6.1c (sample images mounted between the WR2.2 waveguide). The device under test (DUT) (here it is Si-VPC chip) is removed for referencing the measured spectra of samples (see Fig. S6.1c).

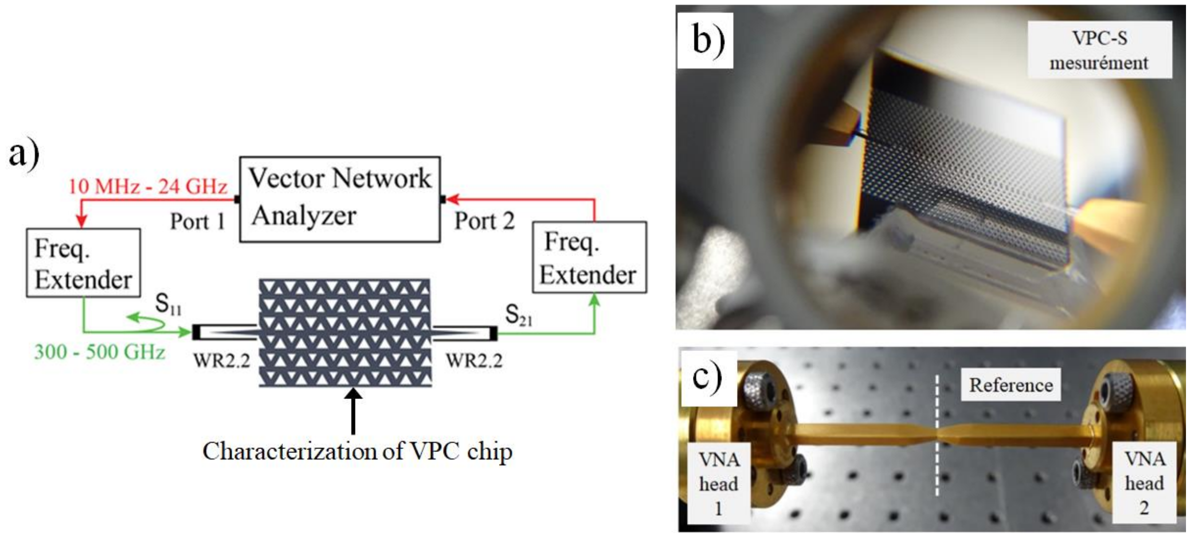

Fig. S6.1: a. Block diagram representation of experimental setup for THz communication experiment **b.** Magnifying image of VPC-S chip. A magnifying lens was used to align the VPC-S chip with VNA waveguiding heads. **c.** Image showing WR2.2 hollow waveguides without the VPC chip for reference measurement.

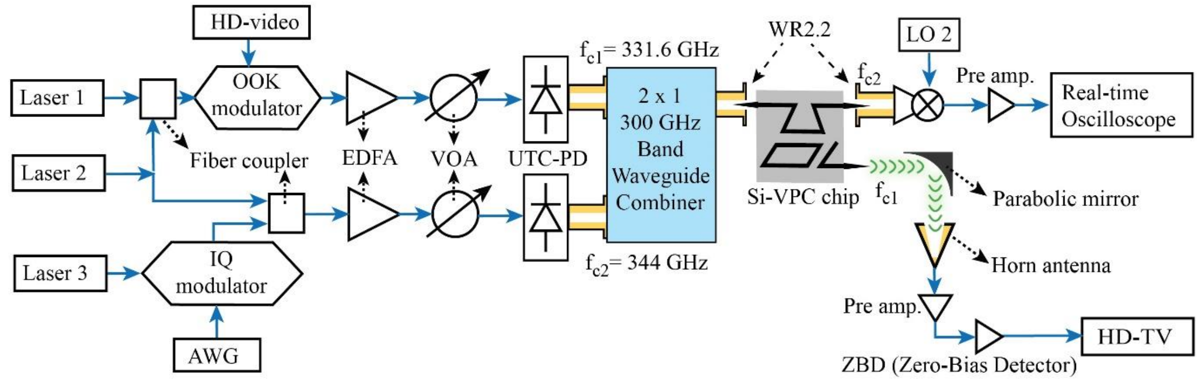

Fig. S6.2: Block diagram representation of experimental setup for THz communication experiment.

Information transfer at an extremely high data rate is one of the attractive features of THz communication. Figure S6.2 shows the block diagram of the hardware setup used in the communication experiment. Two pairs of laser sources (1&2, 2&3) were coupled to generate two independent carrier frequencies. Lasers 1 and 3 were modulated using ON/OFF keying (OOK) and IQ modulators, respectively, to imprint HD-video signal and complex data signals

from arbitrary waveform generator (AWG). The optical signal of coupled laser pairs was amplified by an erbium-doped fibre amplifier (EDFA) and then passed through a voltage-controlled attenuator. The resulting optical signal is fed to a Uni-Traveling-Carrier Photodiode (UTC-PD) to generate 331.6 GHz and 344 GHz carrier frequencies for THz communication. Two channels were combined by a 2 × 1 channel mixer and routed towards the WR 2.2 hollow waveguide. A tapered coupler of Si-VPC chip is used to in-couple and out-couple the THz waves. The demultiplexer signal from two output port tapers was interfaced to the sub-harmonic mixer (SHM) on the receiver side, where a local oscillator (LO1, LO2) was used to retrieve the baseband signal. Later, the amplified signal from CH 2 and CH 1 was visualized using a real-time oscilloscope and HD-TV, respectively.

S7: I-Q intensity diagram

I-Q intensity diagram quantifies the quality of signal recovered at the detector end. It shows the detected symbol after channel equalization and prior to symbol de mapping into data bits. Fig. S8a and S8b show the I-Q intensity constellation diagram for recovered symbols for 160 Gbit/s through VPC-S chip and reference (without the chip) using QAM-32 with 32 GBaud symbol rate. Fig. S8c and S8d show the I-Q intensity constellation diagram for recovered symbols for 125 Gbit/s through VPC-B chip and reference (without the chip) using QAM-32 with 25 GBaud symbol rate. The clearly defined constellation points through both the Si-VPC chip (VPC-S and VPC-B) show the sufficient signal-to-noise (SNR), system fidelity, and good performance of software-based digital-signal processing (DSP) equalization. The highest data rate tested was a 32 GBaud signal with QAM-32 modulation format, corresponding to 160 Gbit/s data rate single channel. The reference signal corresponding to 160 Gbit/s was obtained with standard output powers (-13 dBm at UTC-PD output), while at the output of the VPC-S chip, the system was not able to recover the I/Q maps for 32 GBaud as a signal to noise ratio (SNR) was too low. However, by pushing the testing system to the limits (highest emitted power, featuring -7 dBm at UTC-PD waveguide output, which was 6 dB higher than the normal power level for testing, it was possible to recover the I/Q map using the VPC-S chip, with decent EVM value of 8.2 %. Table S1 shows the EVM and BER values corresponding to Fig. S8a and S8b.

Table S1: EVM and BER value corresponding to VPC-S and reference (without VPC-S chip) for 160 Gbit/s using QAM-32 with 32 GBaud symbol rate.

Device	EVM	BER
VPC-S (Fig. S8a)	8.2	1.3E-2
Reference (Fig. S8b)	7.6	6.5E-3

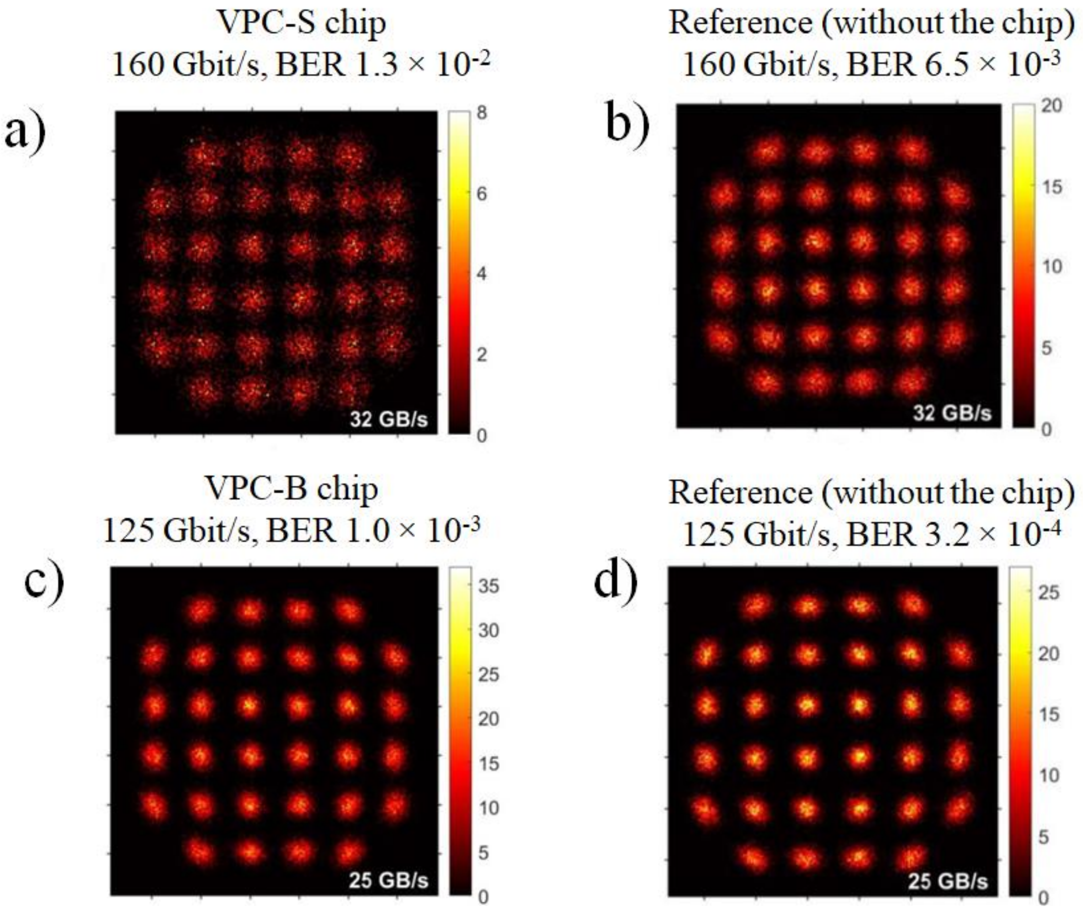

Fig. S8. I-Q constellation diagram for recovered symbols a, b. 160 Gbit/s using QAM-32 (32 GBaud) through VPC-S chip and reference (without the chip). **c, d** 125 Gbit/s using QAM-32 (25 GBaud) through VPC-B chip and reference (without the chip).

In addition, we present the key data communication performance metrices for VPC-S and VPC-B chip in table S2 and S3, respectively.

Table S2: Key data communication performance results of straight (VPC-S) topological chip.

**Waveguide type**	**Data rate**	**Modulation**	**Symbol rate**	**BER**
VPC-S	120 Gbit/s	QAM-16	30 GBaud	4.7×10^{-5}
	125 Gbit/s	QAM-32	25 GBaud	9×10^{-4}
	150 Gbit/s	QAM-32	25 GBaud	5×10^{-3}
	**160 Gbit/s**	**QAM-32**	**32 GBaud**	$\mathbf{1.4 \times 10^{-2}}$

Table S3: Key data communication performance results of bent (VPC-B) topological chip.

Waveguide type	Data rate	Modulation	Symbol rate	BER
VPC-B	100 Gbit/s	QAM-16	25 GBaud	9.2×10^{-7}
	100 Gbit/s	QAM-32	20 GBaud	8×10^{-5}
	**125 Gbit/s**	**QAM-32**	**25 GBaud**	$\mathbf{1 \times 10^{-3}}$

S8: Comparison of transmission spectra from VPC-S and VPC-B chip

Fig. S9 shows the transmission spectra recorded through VPC-S and VPC-B chips. We observe a large bandgap extending from 330 to 355 GHz for VPC-S and VPC-B chips. The availability of such large bandwidth is the key to achieve a high data rate from the Si-VPC chip. However, we observe a small dip at 325 GHz in the transmission spectrum of the VPC-B chip. This effectively reduces the bandwidth for the communication channel, limiting the highest data rate for the VPC-B chip to be at 125 Gbit/s using QAM-32 with 25 GBaud symbol rate (see section S7). We argue that the presence of an additional dip at a lower frequency can be eliminated by optimizing the coupler design of the VPC-B chip.

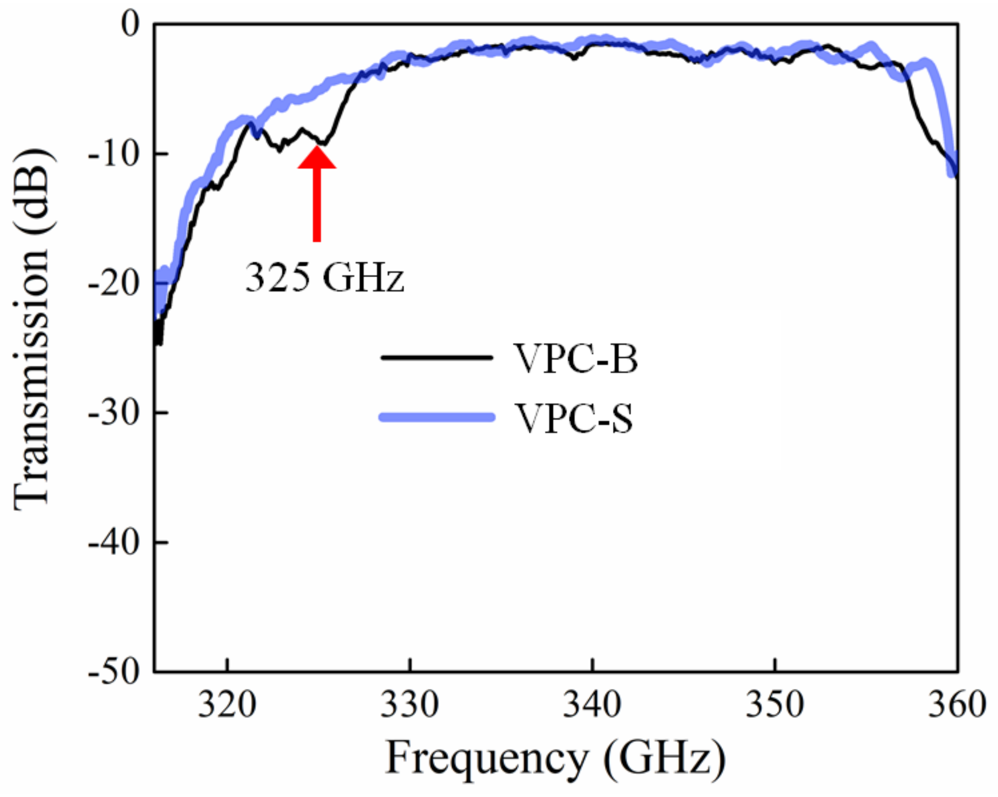

Fig. S9 Comparison of transmission spectra for VPC-S and VPC-B chip

S9: Optical images of Si-VPC chip

Fig S10 shows the optical image of the VPC-B chip, which includes a Z- shaped domain wall to highlight the robustness of THz propagation. The tapered coupler is designed to efficiently in and out couple the THz waves to the VPC-B chip. The waveguiding path is highlighted with a blue shaded region.

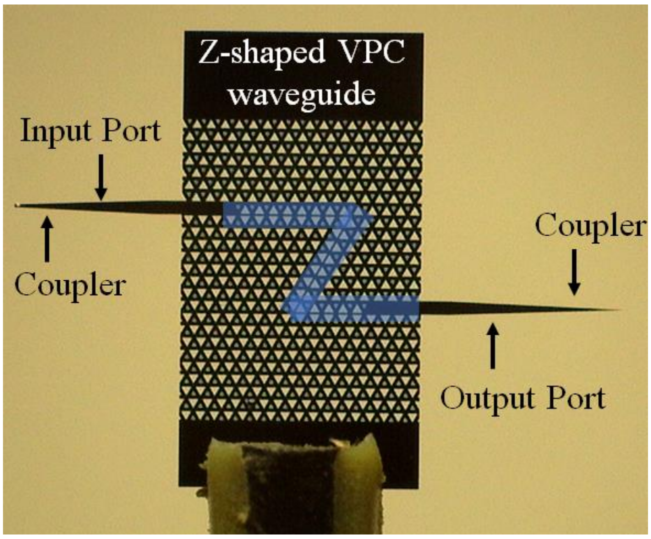

Fig. S10 Optical image of VPC-B chip

S9: Reason behind 0.4 % shift in the topological cavity resonance frequency in the demultiplexer experiment

We performed extensive additional experiments to identify the possible sources of 0.4 % shift in carrier frequency. We identified three possibilities:

1. Modification in overall frequency response dispersion (topological demultiplexer chip + Out coupling effect using a parabolic mirror).
2. Dispersion of the transmitter/receiver.
3. Uncertainty of the wavelength meter that determines the carrier frequency.

1. **Modification in the overall frequency response dispersion (topological demultiplexer chip + out coupling effect using a parabolic mirror)**
   Here, we want to highlight that, the physical separation between the output ports (port 2 and port 3) was not sufficient to feed the output signals directly to the receiver waveguide at the same time as shown in Fig. S11.

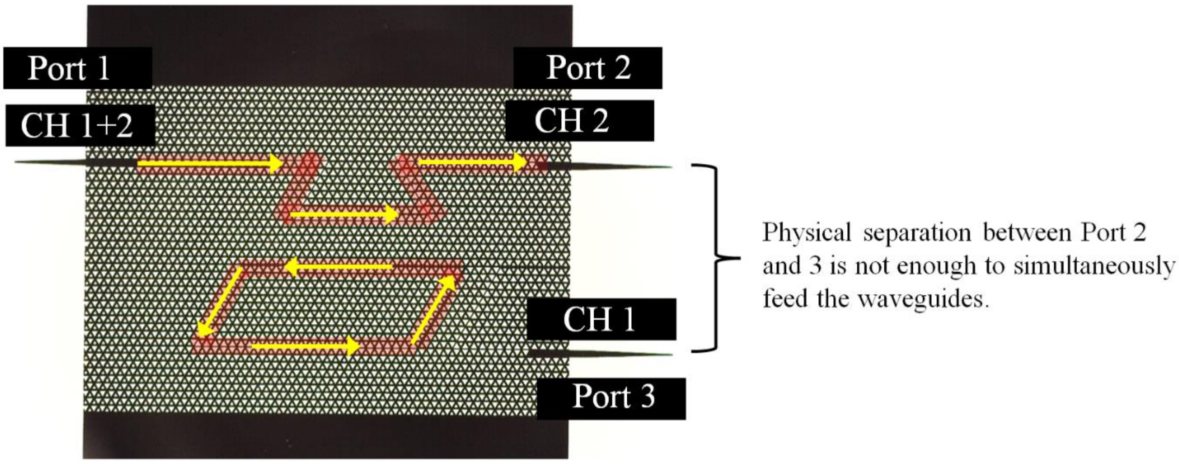

Fig. S11 Topological demultiplexer chip

Since we wanted to showcase the demultiplexing functionality of topological waveguide-cavity chip (Fig. S11) by extracting dual frequency signals simultaneously at the output ports (port 2 and port 3). To circumvent the limitation imposed by the output waveguide tapers of the topological waveguide-cavity chip (see Fig. S11), we extracted the signal from the channel 1 (CH 1, port 3) by inserting a parabolic mirror between the metallic hollow waveguide and waveguide taper (see Fig. S12a).

The experimental setup is shown in Fig. S12a. In this case, a parabolic mirror is used (marked by ‘B’ in Fig. S12b) to collect the THz signal from the taper output (marked as ‘A’ in Fig. S12b), and a waveguide horn antenna (marked as ‘C’ in Fig. S12b) was used to reinject the THz signal into the hollow-core waveguide (marked as ‘D’ in Fig. S12b).

This approach is quite different from direct taper coupling into the hollow-core waveguide and results in additional effects as described below:

a. Additional losses of the overall transmission
b. Introduction of standing waves between the horn (marked as ‘C’ in Fig. S12b) and the taper, resulting in a modification of the overall frequency response.

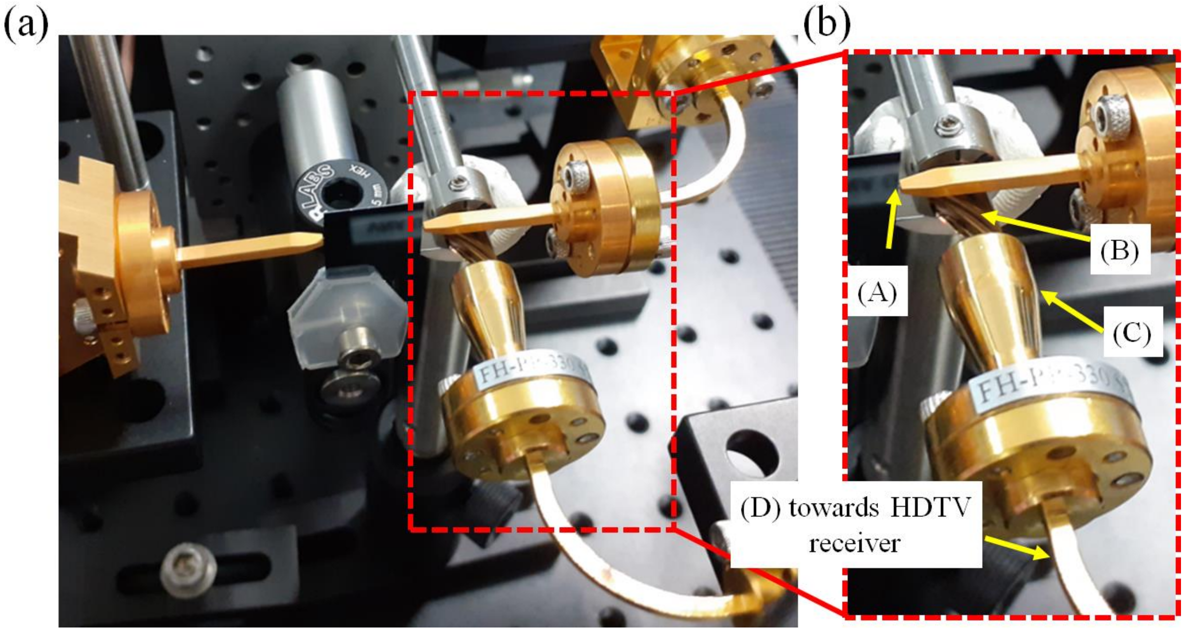

Fig. S12 Experimental setup for THz demultiplexing a. Measurement setup to simultaneously record the data communication from port 2 and port 3 of the topological demultiplexer chip. **b.** Highlighting the receiver arena of the measurement setup. The taper coupler corresponding to port 2 is marked by 'A'. The parabolic mirror used to redirect the data signal via port 3 towards the horn antenna (marked by 'C') is marked by 'B'. The receiver used to collect the HDTV data signal is marked by 'D'.

We investigated the formation of standing waves effects using a vector-network analyzer. Fig. S13 and S14 show the experimental setup for the case of direct waveguide taper coupling (WG-coupling) and free space coupling cases.

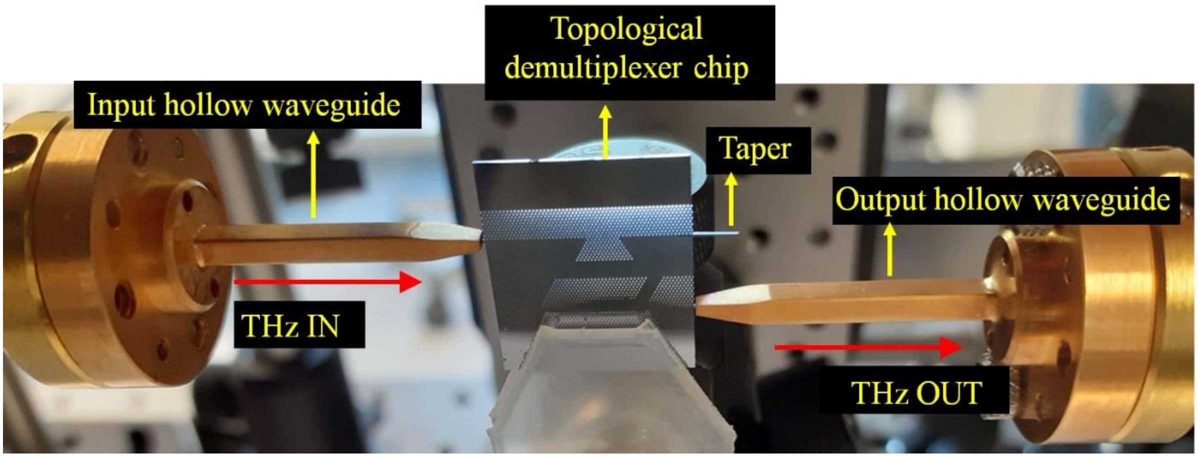

Fig. S13 Experimental setup to measure the transmission through port 3 (CH 1) directly via the waveguide taper coupler from the topological demultiplexer chip.

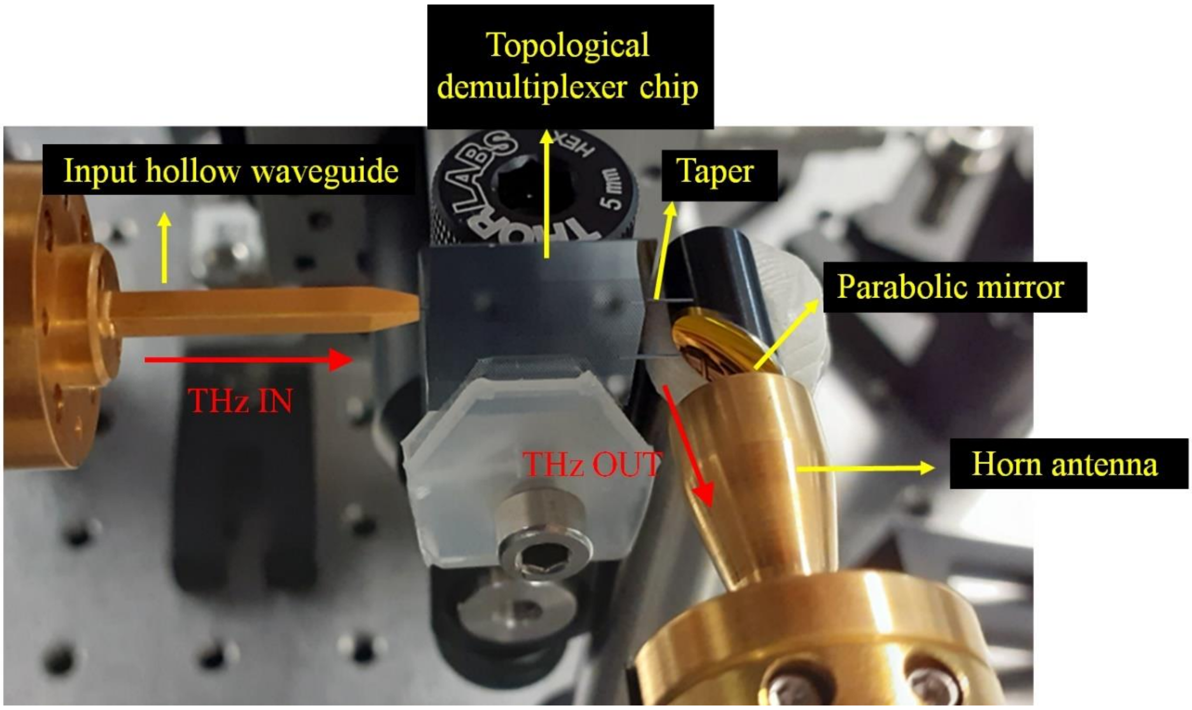

Fig. S14 Experimental setup to measure the transmission through port 3 (CH 1) via free space coupling from the topological demultiplexer chip. For free space coupling, a parabolic mirror is used to redirect the THz signal from the waveguide taper towards the horn antenna.

The transmission spectra corresponding to WG, and free space coupling are shown in Fig. S15. Black square dots (S21) represent the transmission spectrum recorded at output port 2 (see Fig. S15), while blue triangle and red circle show the transmission spectra (S31) recorded via direct WG coupling and free space coupling, respectively. We observed that the free-space coupling induced between 6-10 dB additional losses in the range around the resonance frequency (off set frequency 0 in Fig. S15).

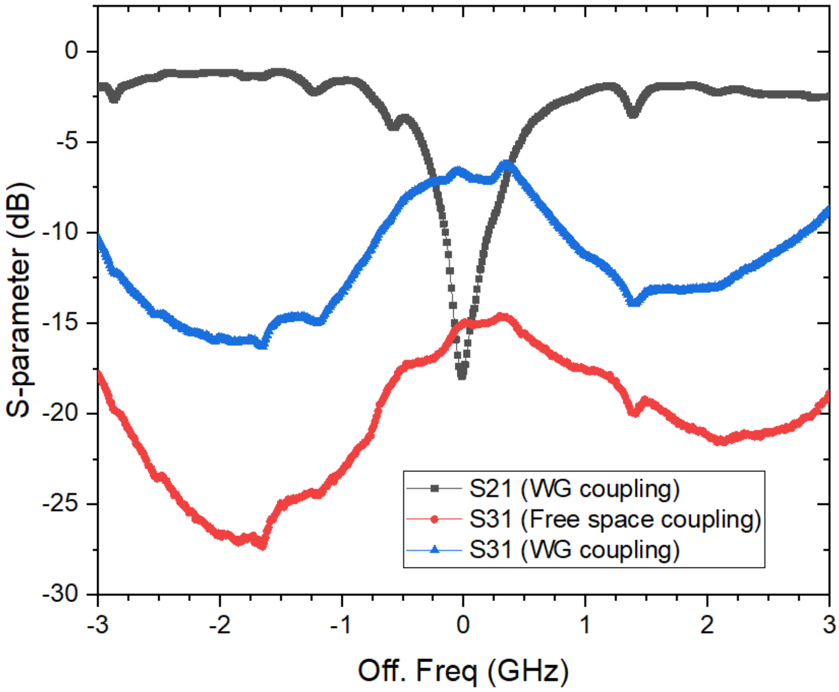

Fig. S15 Transmission spectra recorded from the topological demultiplexer chip. Black square dots show the transmission spectra (S21) recorded through port 2 (see Fig. R6) via direct waveguide taper coupling (WG coupling). The dip in S21 corresponds to the topological cavity's eigenfrequency. Similarly, blue triangles and red circles show the transmission spectra recorded from port 3 (S31) (see Fig. R6) via direct WG coupling and free space coupling, respectively. The offset frequency at x-axis is the frequency difference from resonant frequency.

In addition, we observed change in the transmission spectra when moving the location of the horn antenna at the receiver port. We attribute this change due to the formation of the standing wave between the waveguide taper and horn antenna (see Fig. S16). To illustrate the effect of the position of horn antenna on transmission spectra, we recorded the transmission by varying the position of the horn antenna as shown in Fig. S16a.

Furthermore, to highlight the effect of standing wave produced by the parabolic mirror outcoupling, we plot the evolution of S31 with a moving mirror along the optical axis, shown in Fig. S16b. We observed, peak-to-peak 1.5 dB fluctuation at the resonance frequency (@ 330.2 GHz) (black dots in Fig. S16b) and 3 dB fluctuation with 1.4 GHz (@ 331.6 GHz) resonance frequency offset (red dots in Fig. S16b). The evolution of S31 was extracted by taking the peak value of S31 from Fig. S16a (black and blue dashed vertical lines).

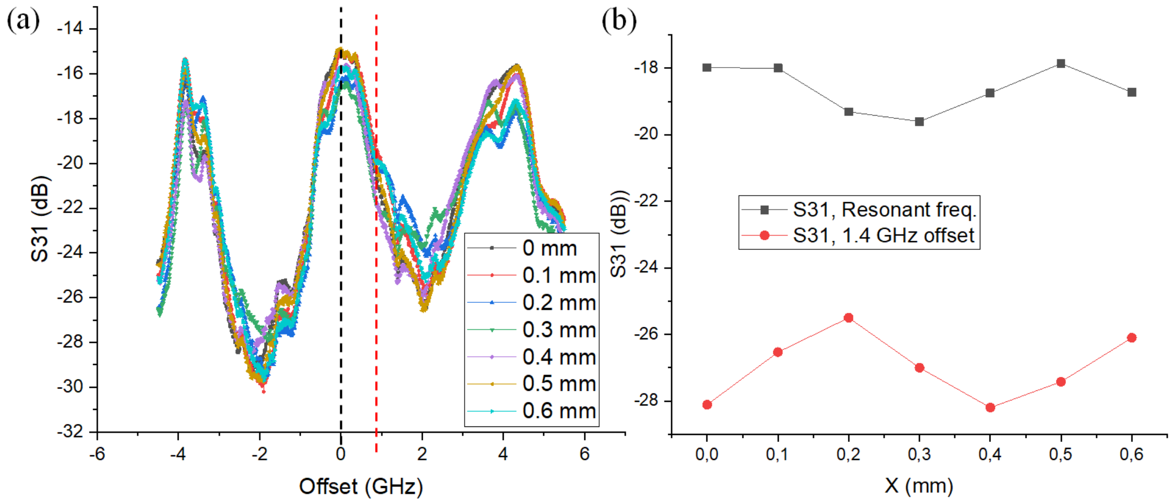

Fig. S16 Evolution of S31 as a function of parabolic mirror position (X) to showcase the formation of standing wave between the horn antenna and the parabolic mirror. a. S31 recorded by varying the position of the parabolic mirror. Black vertical line highlights the resonance frequency (330.2 GHz) of the topological cavity, while red vertical line represents the frequency (1.4 GHz offset from the resonance frequency, 331.6 GHz) used to send the HDTV signal to CH 1 (port 3). The offset frequency at x-axis is the frequency difference from resonant frequency. **b.** The evolution resonance (black square dots) and offset (red circle dots) frequency in S31 as a function of parabolic mirror position (X).

At 330 GHz, the wavelength is 0.9 mm, and the evolution of S31(X) as a function of mirror position (X) shows a periodic behaviour with periodicity close to 0.45 mm (see Fig. RS16b, black dots). It confirms that the presence of standing waves induced by the presence of the parabolic mirror.

2. Dispersion of the transmitter/receiver

During the experiments, the HDTV channel was using a uni-travelling carrier photodiode (UTC-PD) as a source, and a combination of direct detection/amplifier as a receiver. We observed that, the UTC-PD and amplifier featured a flat bandwidth response. However, the direct detection provided by the GaAs Schottky barrier (Zero Barrier Diode, ZBD) diode was not flat.

We checked the frequency response of ZBD using a multiplication chain, a power meter and the ZBD. After the power calibration (PM5 VDI), we measured the output voltage generated by the ZBD and the result is shown in Fig. S17.

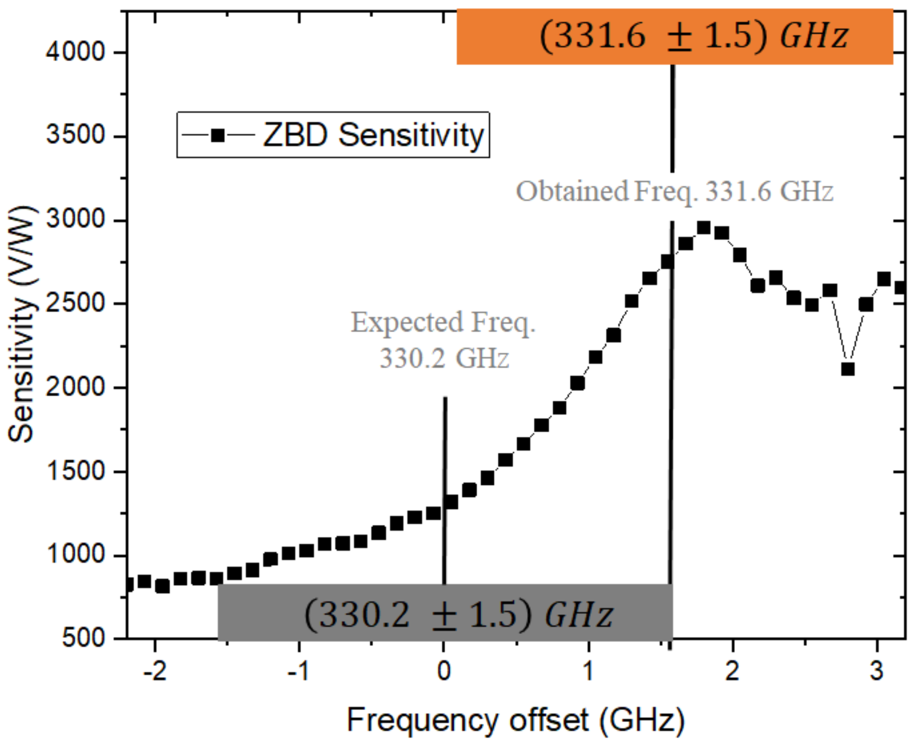

Fig. S17 Frequency response of GaAs Schottky barrier (Zero Barrier Diode, ZBD) diode. The frequency offset at x-axis is frequency difference from resonant frequency. Gray shaded region highlights the $\pm 1.5\ GHz$ bandwidth region around 330.2 GHz carrier frequency, while orange shaded region mark the $\pm 1.5\ GHz$ bandwidth region around 331.6 GHz carrier frequency.

Since we used HDTV signal with 1.5 Gbit/s using amplitude modulation, ON-OFF key (OOK). Therefore, we estimated the channel bandwidth to be around $\pm 1.5\ GHz$ around carrier frequency, as highlighted by grey and orange shades in Fig. S17.

Fig. S17 reveals that over the modulated signal bandwidth, the sensitivity of ZBD detector is higher at 331.6 GHz. Furthermore, the ZBD exhibits less distortion at 331.6 GHz which is better for detection of any modulated signal.

Thus, we optimized the position of the parabolic mirror so that a frequency shift was found in the optimal operational frequency region of ZBD detector. Therefore, we used 331.6 GHz as a carrier frequency to obtain the better performance imposed by the overall communication testing system (mainly dominated by ZBD frequency response), not from the topological demultiplexer chip.

3. Uncertainty of the wavelength meter that determines the carrier frequency

Last, we checked the accuracy of the wavelength meter (86120B from Agilent/Keysight) used in our experiment. The wavelength meter has wavelength accuracy ± 3 ppm ($\pm 0.005\ nm$ at 1550 nm) for laser lines separated by > 30 GHz.

In our case the separation of laser lines was > 300 GHz. Therefore, the maximum inaccuracy was then 0.005 nm corresponding to 0.62 GHz. Thus, the error in the determination of carrier frequency is less than 0.62 GHz.

In conclusion, we believe that the combination of all three effects discussed above, can explain the 0.4 % apparent shift (~ 1.6 GHz) observed during the measurement of modulated HDTV signal (CH 1).

S10: Theory of critical coupling in the topological waveguide-cavity system:

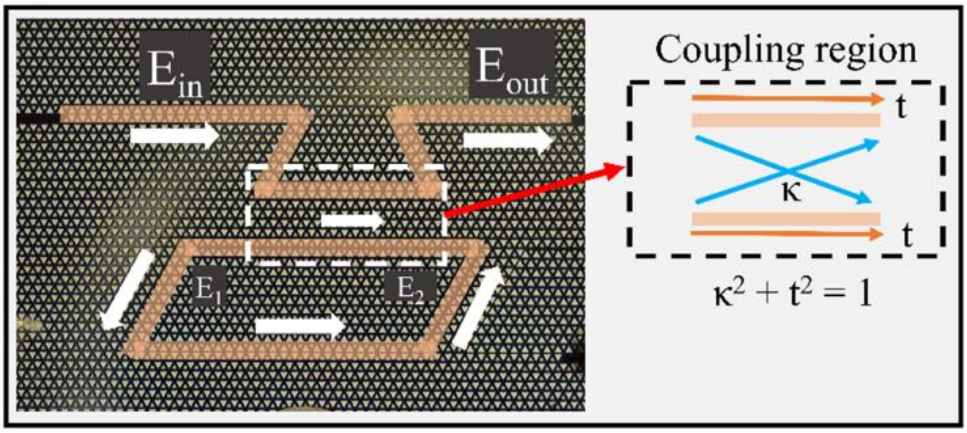

Fig. S18 Coupling mechanism in the topological waveguide-cavity chip.

Fig. S18 shows the coupling mechanism between the topological waveguide and cavity, where t and κ are the transmission coefficient and coupling coefficient, respectively. E_{in} and E_{out} are the input and output electric fields respectively. The incident light couples from the topological waveguide to the topological cavity via coupling region as highlighted in Fig. S18. The field at the input facet of the coupling regime (E_1) becomes E_2 after the round trip in the topological cavity and related by the following relation:

$$E_2 = e^{-2\pi L\alpha}\, e^{i\phi} E_1 = \; re^{i\phi} E_1 \qquad (S2)$$

where, $r = e^{-2\pi L\alpha}$ is the round-trip factor of the topological cavity which accounts for the loss indued during each round trip of wave inside the topological cavity of length L and attenuation factor α. While ϕ is the round-trip phase. Here, we want to highlight that the photoexcitation allows to tune the attenuation (α) of the topological cavity which is directly proportional to the power (P) of the photoexcitation, $\alpha \; \propto P$.

The input and output fields are related as:

$$\begin{pmatrix} E_{out} \\ E_1 \end{pmatrix} = \begin{pmatrix} t & \kappa \\ -\kappa^* & t^* \end{pmatrix} \begin{pmatrix} E_{in} \\ E_2 \end{pmatrix} \qquad (S3)$$

From Eq. S2 and S3 the magnitude of transmission dip (T) can be calculated as:

$$T = 1 - \left(\frac{E_{out}}{E_{in}}\right)^2 = 1 - \frac{(t-r)^2}{(1-ra)^2} \qquad (S4)$$

here, we used the following assumptions: $t^2 + \kappa^2 = 1$, $t = t^*$ and $\phi = 2n\pi$ for constructive coupling the fields from the topological cavity to the waveguide, where n is an integer.

Eq. S4 represents the coupling state of the topological cavity-waveguide system, determined by t and r. For a fixed waveguide and cavity, distance t is constant, while r can be changed by an active way such as photoexcitation. From Eq. S4, the value of T attains maxima at $t = r$ which attests to the critical coupling condition.

Fig. S19 shows the enhancement in transmission dip at cavity resonance frequency upon photoexcitation while maintaining the ultra-high Q factor of the topological cavity. A significant transmission dip ensures high SNR while the high Q factor of the topological cavity provides excellent channel isolation, thus suitable for demultiplexer functionality.

Furthermore, the experimentally measured transmission spectra from the waveguide-cavity chip (Fig. S19) yields the loaded quality factor (Q_{ld}) which follows the following relation:

$$\frac{1}{Q_{ld}} = \frac{1}{Q_{in}} + \frac{1}{Q_c(g)} \qquad (S5)$$

where, Q_{in} is the intrinsic quality factor of the topological cavity, defined as Q factor of the uncoupled topological cavity and $Q_c\ (g)$ accounts for the losses due to waveguide-cavity coupling which depends on the separation (g) between the waveguide and cavity.

Since at critical coupling, the intrinsic loss of the cavity becomes equal to the waveguide-cavity coupling loss, which means $Q_{in} = Q_c(g)$.[3–5] Therefore, Eqn. S5 yields

$$Q_c(g) = 2Q_{ld} \qquad (S6)$$

Hence, using the experimentally measured loaded quality factor (Q_{ld}), we can extract the $Q_c(g)$ and using the relation $Q_c(g) = 1/\gamma_c$ we can evaluate the waveguide-cavity coupling loss (γ_c).

S11: Phototunable critical coupling in the topological cavity:

For data communication perspective, the critical coupling is vital, as it provides a new modality for channel switching, filtering and demultiplexing applications. In addition to switching, photoexciting the topological cavity allows to achieve the critical coupling by fine tuning the intrinsic loss of the topological cavity, as shown in Fig. S19. The attainment of critical coupling manifests as intensity enhancement of the topological cavity resonance. Fig. S19 shows the experimentally recorded transmission spectra. The black dots depict the transmission spectrum measured for pristine (without photoexcitation, power = 0 mW), while solid red line shows the transmission spectra recorded with low power (power = 0.55 mW) photoexcitation. The enhancement in the topological cavity's resonance intensity signifies the critical coupling.

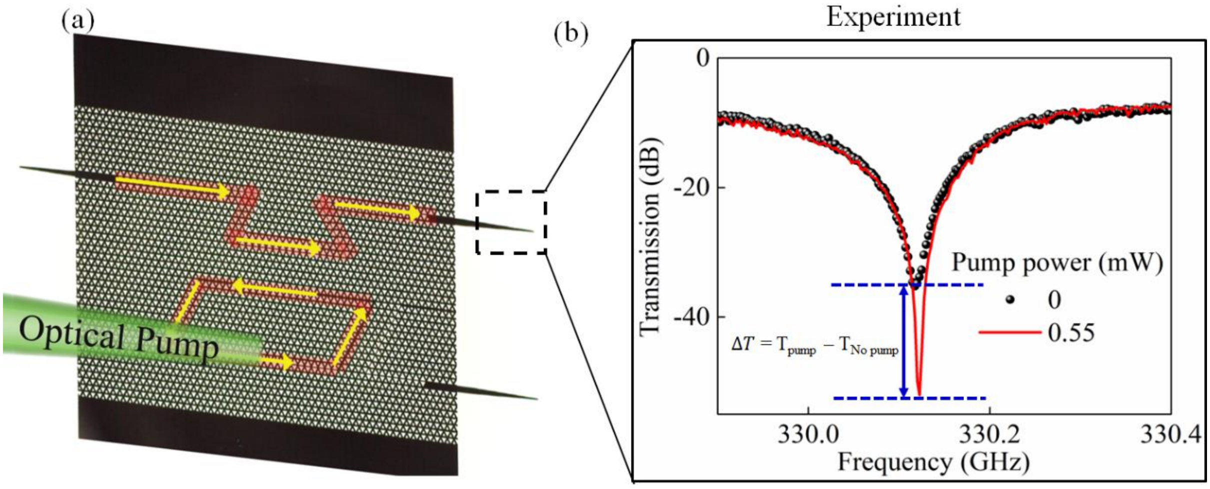

Fig. S19 Dynamic critical coupling a. Mechanism of achieving critical coupling in the topological waveguide-cavity chip via photoexcitation. **b.** Experimentally measured transmission spectra from the output port of the topological waveguide-cavity chip (shown in 'a'). The black dots represent the intrinsic (without photoexcitation, power = 0 mW) transmission spectra while the red solid line represents the critical coupling case (with photoexcitation, power = 0.55 mW).

S12: Effect of photoexcitation in the demultiplexing experiment:

We performed the THz communication (demultiplexing experiment) measurement by photoexciting the cavity channel (CH1). The results are shown in Fig. S20. Fig. S20a shows the THz communication results when the optical pump is absent (PUMP OFF). In this case, the topological cavity performs the channel demultiplexing as result we observe uninterrupted HD-video streaming on HD-TV at CH 1 (lower panel of see Fig. S20a) and 40 Gbit/s data streaming at CH 2. Further, photoexciting the channel near to the topological cavity with 5 mW optical pump, the HD-video streaming via CH1 gets interrupted shown in Fig. S20b, while the 40 Gbit/s data streaming at CH2 is uninterrupted. Further, we recorded a video to clearly the effect of photoexcitation at cavity channel and provided as supplementary video SVc.

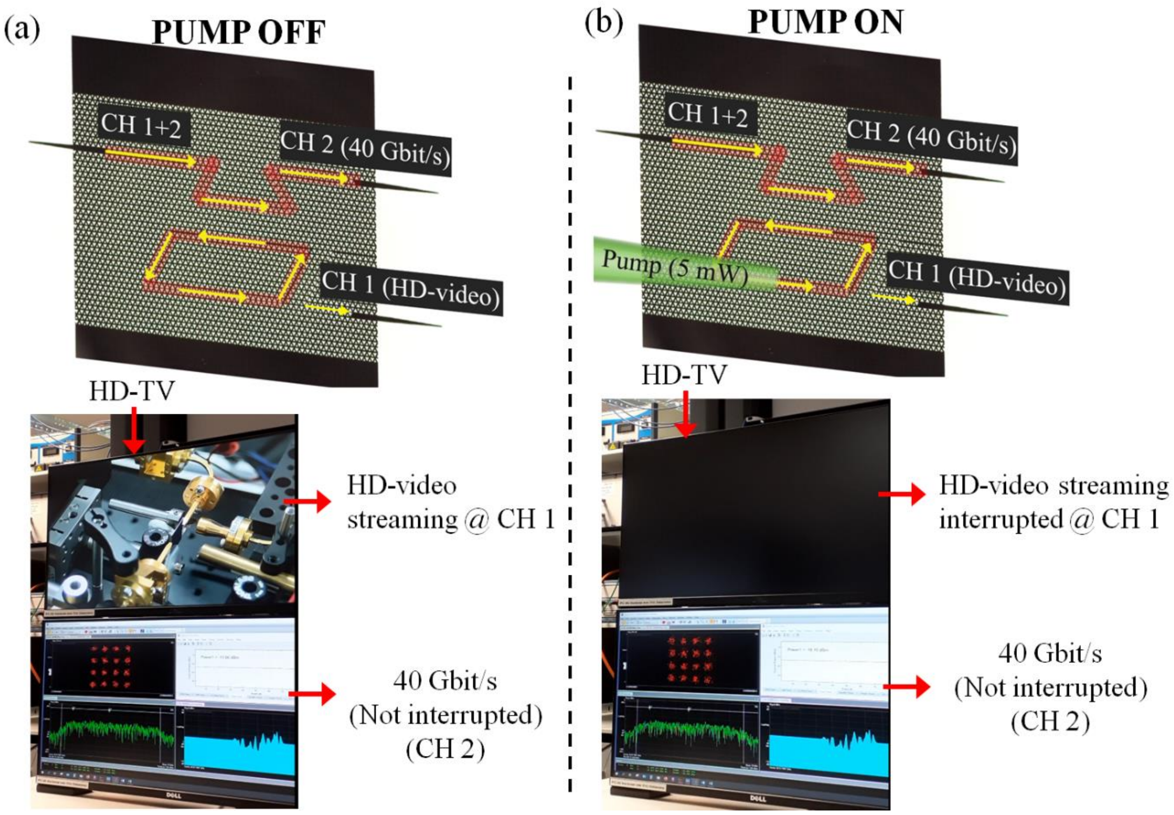

Fig. S20 Phototunable topological demultiplexing a. Pump OFF case. Top panel shows the optical image of the fabricated topological waveguide-cavity chip. At the input facet the data signals at two carrier frequencies ($f_1 = 331.6\,GHz$ and $f_2 = 344\,GHz$) are inserted into the chip that further demultiplexed into two well isolated channels viz. CH1 (HD-Video) and CH2 (40 Gbit/s). The bottom panel shows the HD video streaming at the HD TV streaming via CH1, and I-Q constellation diagram corresponding to 40 Gbit/s data signal. **b.** Pump ON case. The top panel illustrates the photoexcitation at the domain of the topological cavity, which interrupts the HD-Video transmission at CH1. However, the 40 Gbit/s data transmission via. CH2 is not interrupted, shown in the bottom panel.

S13: Optical images of the fabricated topological device

To showcase the state-of-the-art fabrication quality below we show the optical images of the fabricated topological devices. The salient features of our fabrication are following:

1. **Uniform and simultaneous etching** of variable aspect ratio structures (1:5).
2. **Sharp corners** of triangular etch holes with **extremely straight silicon sidewalls** (> 89 deg) for 200 μm thickness as shown in Fig. R15b.
3. 100 % fabrication yield and repeatability of the process, verified across foundry level 8-inch silicon wafers.

<u>Full optical images of the fabricated topological device:</u>

Fig. S21, S22 and S23 show the optical images of the straight, Z-bend and waveguide-cavity coupled fabricated topological device.

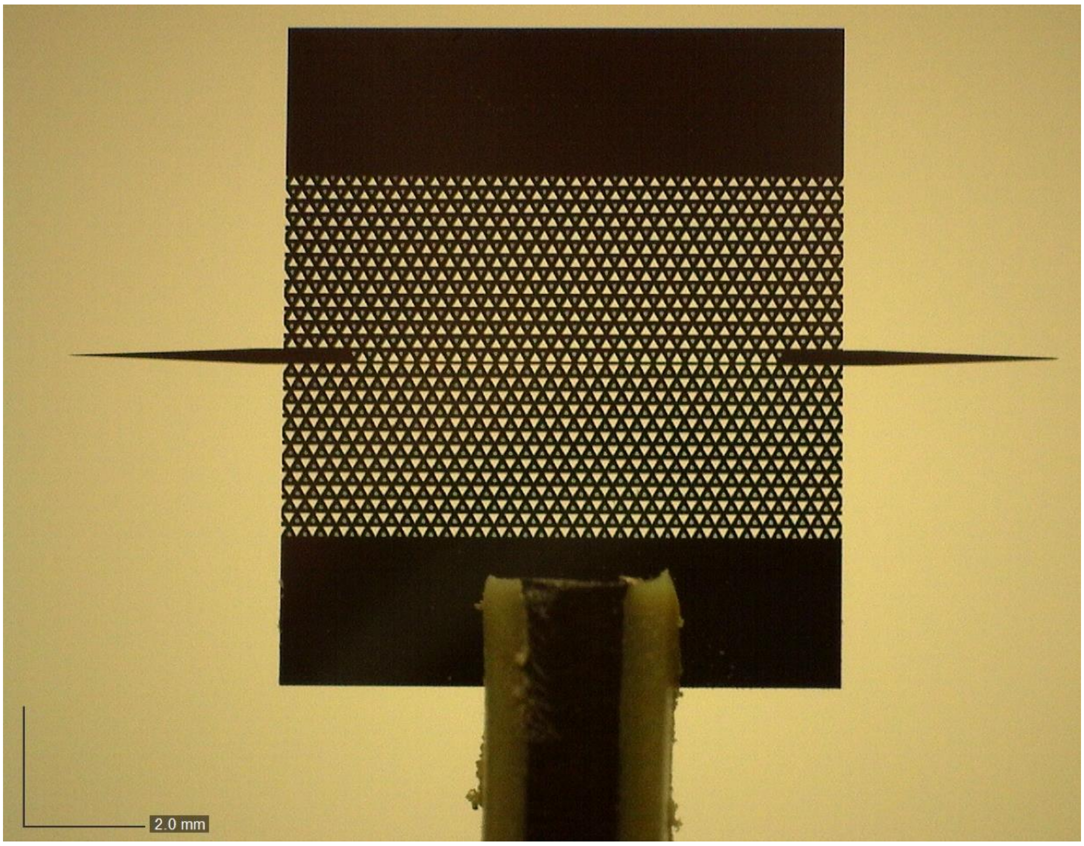

Fig. S21 Optical image of the straight topological waveguide.

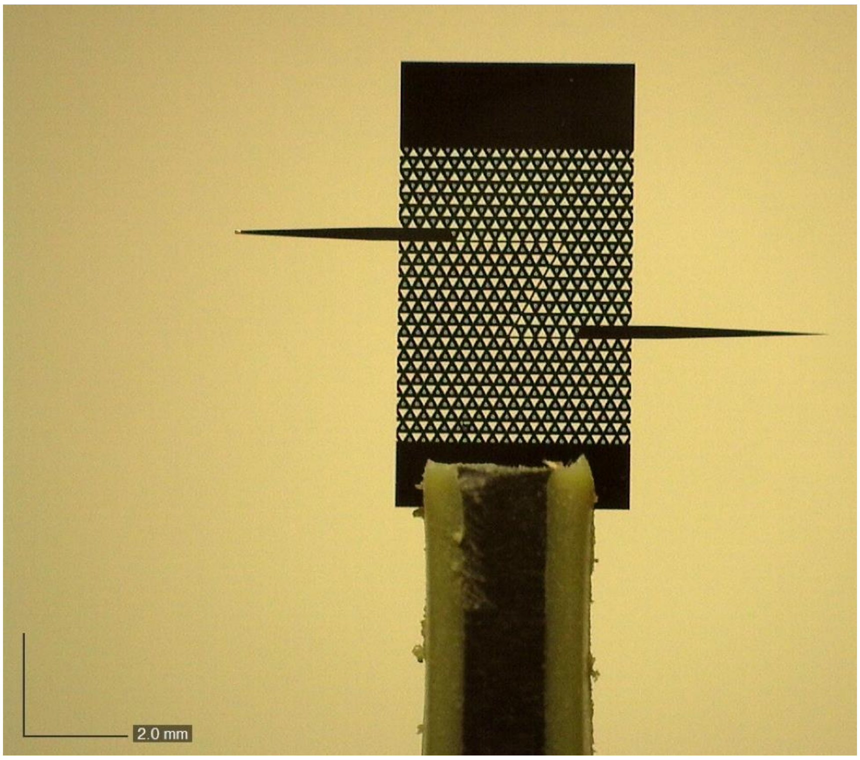

Fig. S22 Optical image of the bend (Z-shaped) topological waveguide.

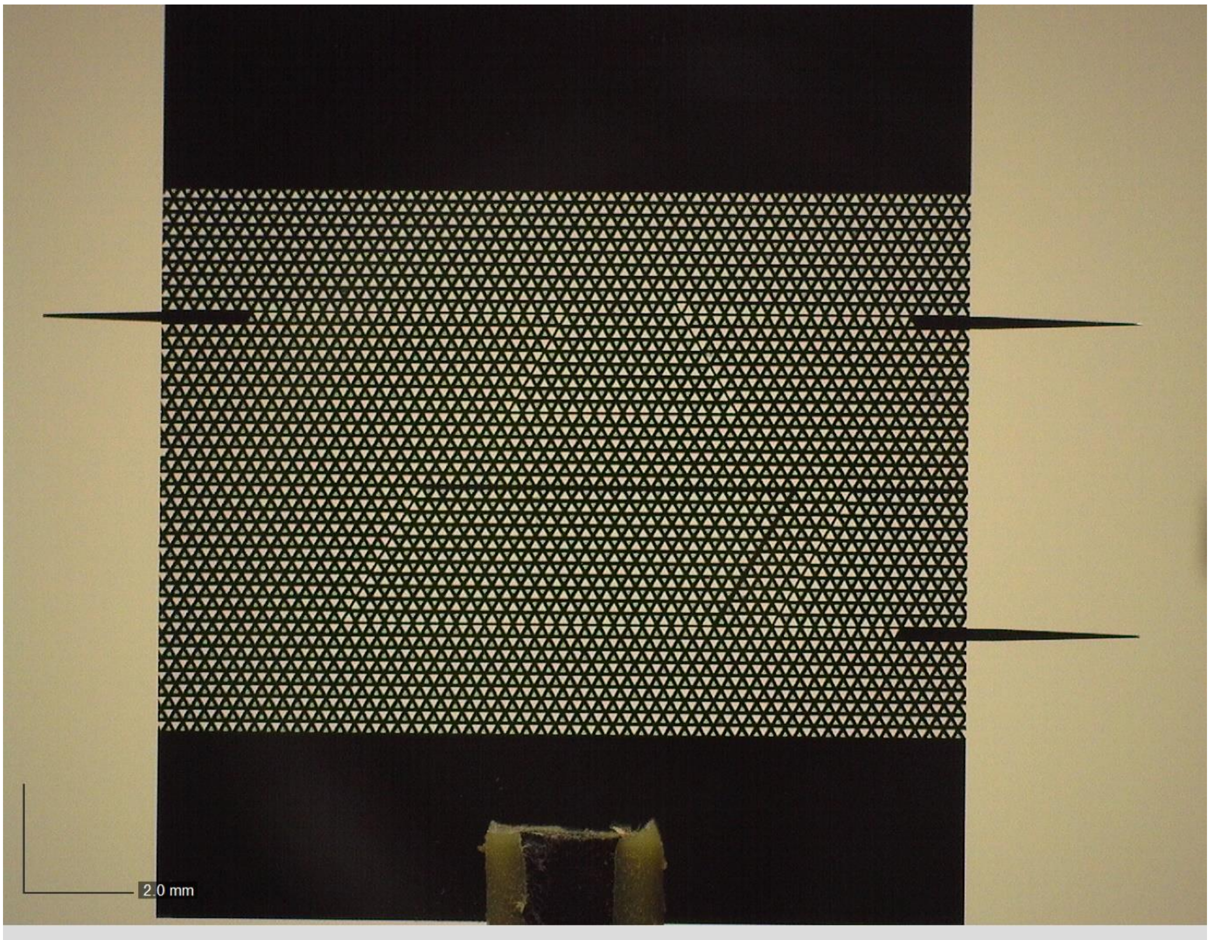

Fig. S23 Optical image of the waveguide-cavity integrated topological demultiplexer chip.

Optical images of the fabricated topological device zoomed at the coupler region:

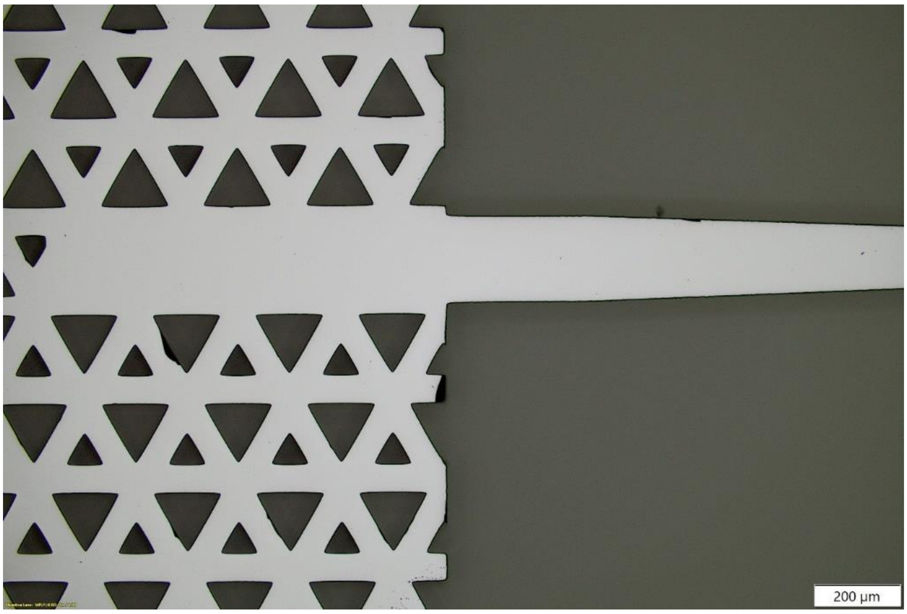

Fig. S24 Optical image of the Si-VPC chip highlighting the coupler area.

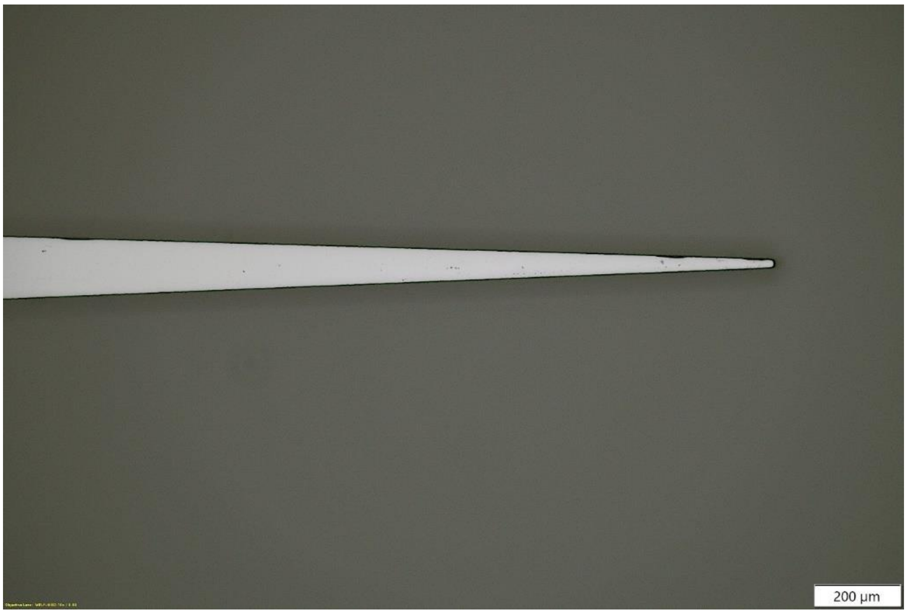

Fig. S25 Optical image of the Si-VPC chip highlighting the coupler.

<u>Scanning electron microscope (SEM) images of the fabricated topological device to showcase the uniform etching and sharp corners of the triangular holes:</u>

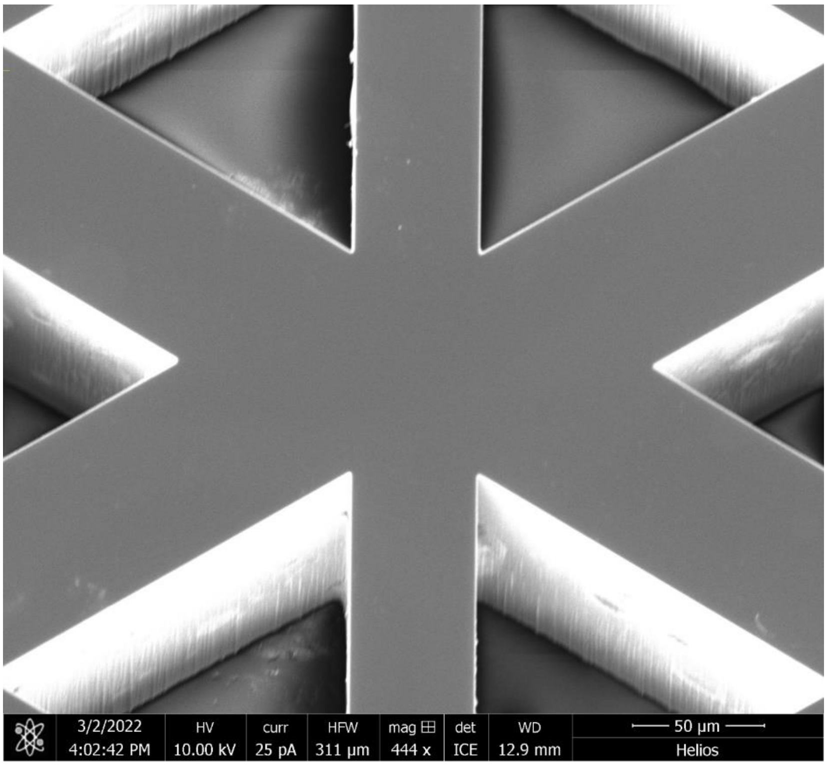

Fig. S26 SEM image of the fabricated Si-VPC chip, clearly illustrating uniform etching profile of the triangular holes.

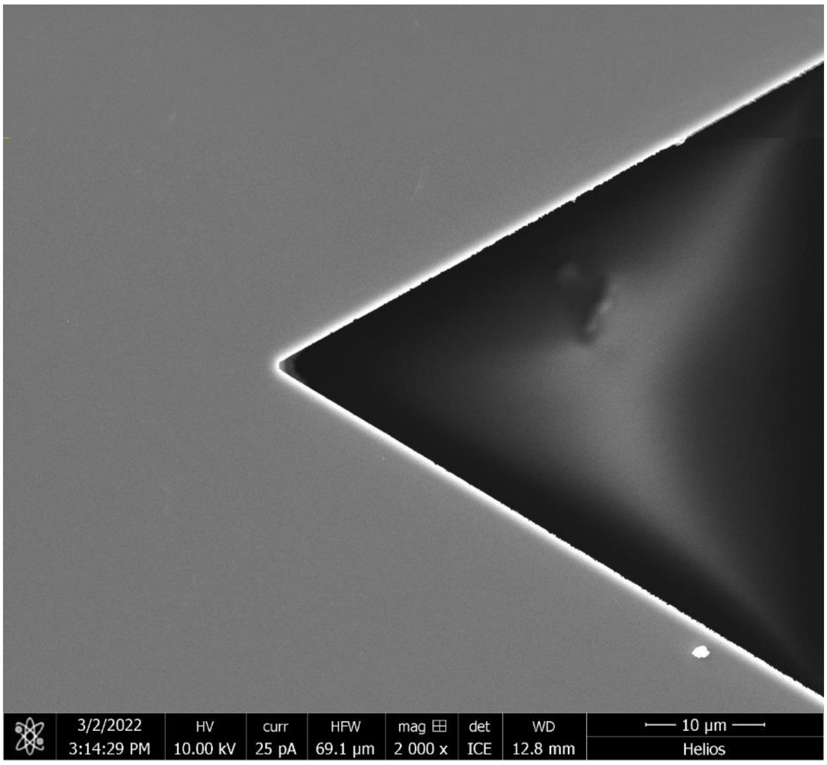

Fig. S27 SEM image of the fabricated Si-VPC chip, clearly illustrating uniform and sharp edges of etched triangular holes.

S14: Simulated transmission spectra from the four ports topological waveguide-cavity configuration

To reroute the signal from the topological cavity we designed a drop waveguide (i.e., port 3), shown in Fig. S28a. Designing the drop waveguide, inherently yields an additional port i.e., port 4 (Fig. S28a) due to the Type A and Type B arrangements of the unit cell. We optimised the design of topological demultiplexer chip such that the existence of an additional port (port 4) does not affect the transmission performance at drop waveguide (CH1 or port 3). To highlight this, we performed the 3D simulation in CST Microwave studio, for the four ports topological waveguide-cavity chip. The simulated results are shown in Fig. S28b, where it is evident that the presence of an additional port (i.e., port 4) does not affect the transmission spectra corresponding to port 2 and port 3 and preserve the demultiplexing functionalities.

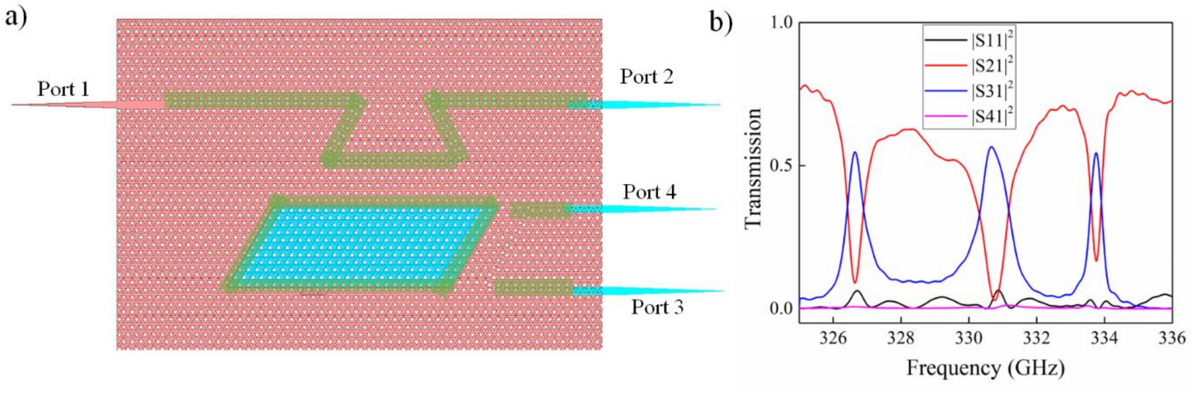

Fig. S28 a. Schematic of topological demultiplexer chip with four ports configuration. **b.** Simulated transmission spectra recorded at each port using 3D simulation in CST Microwave studio.

References:

1. Gong, Y., Wong, S., Bennett, A. J., Huffaker, D. L. & Oh, S. S. Topological Insulator Laser Using Valley-Hall Photonic Crystals. *ACS Photonics* **7**, 2089–2097 (2020).
2. Yu, S.-Y. *et al.* Critical couplings in topological-insulator waveguide-resonator systems observed in elastic waves. *Nat. Sci. Rev.* **8**, nwaa262 (2020).
3. Vanier, F., Mela, C. L., Hayat, A. & Peter, Y.-A. Intrinsic quality factor determination in whispering gallery mode microcavities using a single Stokes parameters measurement. *Opt. Express, OE* **19**, 23544–23553 (2011).
4. Yariv, A. Critical coupling and its control in optical waveguide-ring resonator systems. *IEEE Photonics Technology Letters* **14**, 483–485 (2002).
5. Cai, M., Painter, O. & Vahala, K. J. Observation of Critical Coupling in a Fiber Taper to a Silica-Microsphere Whispering-Gallery Mode System. *Phys. Rev. Lett.* **85**, 74–77 (2000).
