# Supplementary material for: Phototunable chip-scale topological photonics: 160 Gbps waveguide and demultiplexer for THz 6G communication
Source: Nat Commun. 2022 Sep 15;13:5404. doi: 10.1038/s41467-022-32909-6 (PMC9478161; doi:10.1038/s41467-022-32909-6)
Supplement: Supplementary file 2 — Description to Additional Supplementary Information [file 41467_2022_32909_MOESM2_ESM.pdf]

Description of Additional Supplementary Information

Supplementary Movie ‘a’:

High-speed data transmission through silicon topological demultiplexer chip. A real-time wireless streaming of high definition (HD) video through channel 1 (CH 1), while CH 2 supports 40 Gbit/s data transfer speed.

Supplementary Movie ‘b’:

Phototunable topological demultiplexing functionality. Photoexciting the CH 2 switched off the data transmission without affecting the real-time streaming of HD video through CH 1, exhibiting excellent channel isolation.

Supplementary Movie ‘c’:

Photoexciting the CH 1 switched off the real-time streaming of HD video through CH 1 without affecting the 40 Gbit/s data transmission through CH 2, exhibiting excellent channel isolation performance of topological demultiplexer chip.
